# Human Peripheral CD4^+^ Vδ1^+^ γδT Cells Can Develop into αβT Cells

**DOI:** 10.3389/fimmu.2014.00645

**Published:** 2014-12-17

**Authors:** Hendrik Ziegler, Christian Welker, Marco Sterk, Jan Haarer, Hans-Georg Rammensee, Rupert Handgretinger, Karin Schilbach

**Affiliations:** ^1^Department of Hematology and Oncology, University Children’s Hospital, University of Tübingen, Tübingen, Germany; ^2^Department of Immunology, Interfaculty Institute for Cell Biology, University of Tübingen, Tübingen, Germany

**Keywords:** extrathymic T-cell development, Vδ1^+^ γδT cells, T-cell development, δβ heterodimer, inflammation, hematopoietic progenitor cell, extrathymic T-cell progenitor

## Abstract

The lifelong generation of αβT cells enables us to continuously build immunity against pathogens and malignancies despite the loss of thymic function with age. Homeostatic proliferation of post-thymic naïve and memory T cells and their transition into effector and long-lived memory cells balance the decreasing output of naïve T cells, and recent research suggests that also αβT-cell development independent from the thymus may occur. However, the sites and mechanisms of extrathymic T-cell development are not yet understood in detail. γδT cells represent a small fraction of the overall T-cell pool, and are endowed with tremendous phenotypic and functional plasticity. γδT cells that express the Vδ1 gene segment are a minor population in human peripheral blood but predominate in epithelial (and inflamed) tissues. Here, we characterize a CD4^+^ peripheral Vδ1^+^ γδT-cell subpopulation that expresses stem-cell and progenitor markers and is able to develop into functional αβT cells *ex vivo* in a simple culture system and *in vivo*. The route taken by this process resembles thymic T-cell development. However, it involves the re-organization of the Vδ1^+^ γδTCR into the αβTCR as a consequence of TCR-γ chain downregulation and the expression of surface Vδ1^+^Vβ^+^ TCR components, which we believe function as surrogate pre-TCR. This transdifferentiation process is readily detectable *in vivo* in inflamed tissue. Our study provides a conceptual framework for extrathymic T-cell development and opens up a new vista in immunology that requires adaptive immune responses in infection, autoimmunity, and cancer to be reconsidered.

## Introduction

Hematopoietic stem-cells (HSCs) are rare, phenotypically and functionally diverse cells that can give rise to all cell lineages of the immune system (1). T-cell development commences when bone-marrow-derived HSCs seed the thymus. They are the most immature progenitors and thus constitute the CD4^−^CD8^−^ double negative (DN) T-cell fraction. Stroma- and thymocyte-derived signals then induce their T-cell lineage commitment and the cells’ differentiation into either αβ or γδT cells through well-defined stages (DN1–DN4). In humans, these stages can be recognized by the expression of CD34, CD38, and CD1a surface proteins. The expression of functionally rearranged TCR-γ and TCR-δ chain genes in DN2/3 thymocytes leads to γδTCR complexes, which drive cellular proliferation and promote differentiation into γδT cells (2, 3). In order to become an αβT cell, developing DN3 thymocytes need to express functionally rearranged TCR-β chain genes that associate with pre-Tα molecules to form pre-TCR complexes. The pre-TCR signal drives proliferation, induces transcriptional silencing of the TCR-γ chain (4) and initiates the transition of the T cells into CD4^+^ and CD8^+^ expressing double-positive (DP) stages. In humans, this transition involves immature single-positive (ISP) CD4^+^ intermediates (5). DP T cells initiate the rearrangement of TCR-α genes, which leads to the deletion and thus “silencing” of the TCR-δ chain because the genes encoding the TCR-δ chain are embedded in the TCR-α locus (6–10). TCR-α and -β chains form αβTCRs, which are selected for their ability to recognize peptide-presenting self-MHC molecules (positive selection). In this repeated process, cells that carry non-functional TCRs undergo TCR-α rearrangement (11) until selected (2). DP T cells that recognize self-MHC class I or II molecules below an acceptable threshold of reactivity (negative selection) develop into single-positive (SP) CD4^+^ or CD8^+^ αβT cells, and are exported from the thymus into the periphery.

It is undisputed that the thymus provides the foremost source of naïve T cells and orchestrates normal T-cell lymphopoiesis to some degree throughout life (12, 13). However, thymic involution begins as early as 1 year after birth, resulting in an exponentially decreasing output of naïve T cells, which is almost completely extinguished post adolescence (14). The total size of the T-cell pool nevertheless remains relatively constant throughout life (14, 15), which suggests that the T-cell pool must be replenished in some other way. The decreasing number of naïve T cells is in part balanced by the proliferation of peripheral, post-thymic T cells, including naïve(16) and memory αβT cells(16–18), γδT cells(19, 20), and NKT cells(21), leading to effector or long-lived memory T cells (22–24). Moreover, there is a growing body of evidence that suggests that T cells may develop at extrathymic sites in mice (25) and in humans, e.g., in tonsils (26), lymph nodes, spleen, and the bone marrow (27–30). However, detailed knowledge about the precursors, site, and routes of extrathymic T-cell development is still elusive.

Recent research indicates that HSC – generally present in a dormant state in a specialized niche in the bone marrow – can be induced to proliferate and differentiate under conditions of stress (31–33). It has also been shown that they respond to T-cell consumption by inducing the proliferation of common lymphoid progenitors (CLPs), which are the immediate progenitors of T cells (31–33). Vδ1^+^ γδT cells are key players in the lymphoid stress-surveillance response. They constitute a minor T-cell population in the peripheral blood, but a major subset among tissue-residing and intraepithelial lymphocytes (34–37).

In this study, we show that the rare and so far unappreciated entity of human CD4^+^ Vδ1^+^ γδT cells, isolated from the peripheral γδT-cell pool of healthy individuals, expresses markers that are characteristic of the earliest hematopoietic progenitor cells, i.e., multipotent (MPP) and CLPs. Like thymus-seeding, early T (ETP), and DN1 progenitors, CD4^+^ Vδ1^+^ γδT cells express CD34 and CD38 but not CD1a (CD34^+^ CD38^+^ CD1a^neg^) on their surface; they also carry full-length transcripts of in-frame δ, γ, and β TCR gene rearrangements and express recombination-activating gene (RAG) and terminal deoxynucleotidyl transferase (TdT), which are typically found in DN2 and DN3 thymocytes. We show that CD4^+^ Vδ1^+^ γδT cells that lack thymus-homing properties but carry chemokine receptors (CCR) that direct circulating T cells to sites of inflammation, can develop into functional, mature CD4^+^ or CD8^+^ αβT cells in an inflammatory environment. In this study, we pinpoint the individual steps of this development, a process that is very similar to thymic T-cell development, but proceeds via a Vδ1^+^Vβ^+^ intermediate instead of a pre-TCR. We also show that the progenitors’ cellular intermediates are present *in vivo* in inflamed tissue and to a considerably lesser extent in peripheral blood of healthy individuals.

This fundamentally new role of γδT cells as an αβT-cell precursor contributes to the emerging concept of T-cell plasticity and recommends the reconsidering of adaptive immune responses in infection, autoimmunity, and cancer.

## Results

### CD4^+^ Vδ1^+^ γδT-cell clones display characteristics of a T-cell progenitor

**Figure 1:**
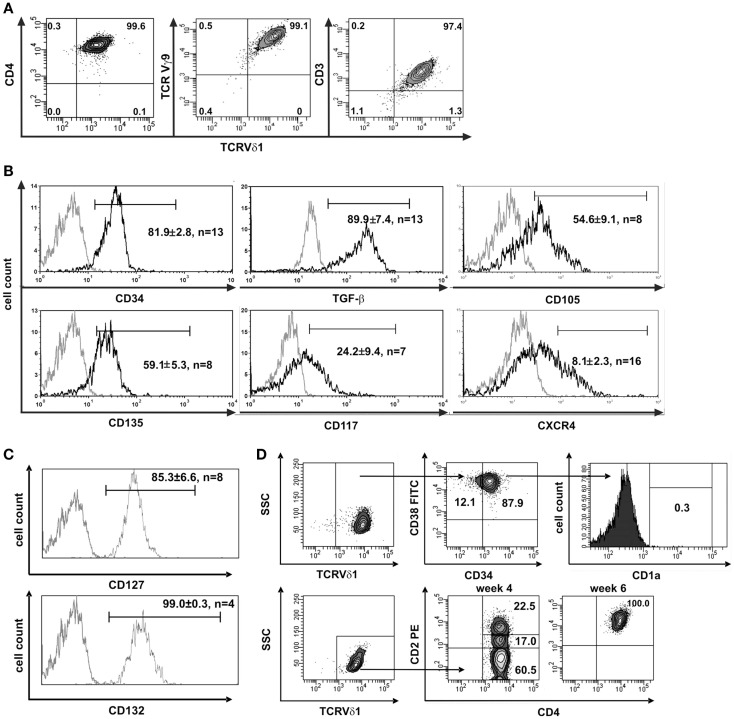
**CD4^+^ Vδ1^+^ cells express hematopoietic stem/progenitor cell markers**. **(A)** CD4^+^ Vδ1^+^ T-cell clone TCRs contain a Vγ9 chain and the cells are CD3^+^. **(B)** CD4^+^ Vδ1^+^ T-cell clones express the stem-cell and progenitor markers CD34, CD135 (FLT3), CD117 (c-kit), CD105 (TGF-βR), and CXCR4 on their surface and express large amounts of TGF-β. Gray line: isotype control. Histogram marker shows cells that stained positive for antigen of interest. Numbers indicate mean ± SEM of CD4^+^ Vδ1^+^ T cells that stained positive for the respective marker (given in %). Each histogram shows one representative experiment of all clones tested. Numbers of clones tested are given in each histogram. **(C)** Vδ1^+^ CD4^+^ T-cell clones express IL-7 receptor composed of α subunit CD127 and the common γ chain CD132 of IL-2R. **(D)** FACS analysis showed that CD4^+^ Vδ1^+^ T-cell clones are CD34^+^CD38^+^CD1a^neg^, may lack CD2 expression, but become CD2^+^ during cultivation.

In this study, we aimed to characterize the scarce T-cell entity of CD4^+^ Vδ1^+^ γδT cells. We generated CD4^+^ Vδ1^+^ γδT clones from the peripheral blood of 12 healthy individuals, from leukapheresis products (LPH) of GM-CSF-mobilized healthy stem-cell donors (*n* = 12), and also from the bone marrow of potential stem-cell donors (*n* = 10). Clones of this phenotype were extremely long-lived – they could be cultivated uninterruptedly *ex vivo* for up to more than a year under standard culture conditions. Importantly, over time, some clones could change their γδTCR into αβTCR. The morphology of the CD4^+^ Vδ1^+^ γδT-cell clones was similar to that of large granular lymphocytes (LGLs) (Figure S1A in Supplementary Material). In contrast to most other Vδ1^+^ cells, their TCR-γ9^+^ chain (Figure 1A) contained a constant-region segment 1 (Cγ1) (Figure S1B in Supplementary Material) and was thus able to form disulfide bonds between TCR-δ and -γ chains (38–40).

To elucidate the nature of the clones’ transdifferentiation from γδ into αβT cells and to clarify whether the change in TCR constitutes a certain form of TCR revision or whether it is the result of progenitor differentiation, clones were examined for the expression of stem-cell and progenitor markers. Although already committed to T-cell lineage (CD3^+^) CD4^+^ Vδ1^+^ γδT-cell clones nevertheless uniformly expressed CD34^lo^ (22/22), which is the common marker of most immature hematopoietic stem/progenitor cells. The clones also expressed C-X-C chemokine receptor type 4 (CXCR4), which maintains the quiescence of the HSC pool in bone-marrow niches (41), TGF-β, a regulator of hematopoietic stem/progenitor cell self-renewal (42–44), and its receptor CD105, which, to some extent, indicates a self-sustaining circuit (Figure 1B). CD4^+^ Vδ1^+^ γδT-cell clones expressed a functional IL-7 receptor (CD127^+^/CD132^+^) (Figure 1C), CD117lo(c-kit) and the FLT3 ligand receptor CD135 (Figure 1B). FLT3 and the CD117-activated signal transduction cascade promote cell survival and proliferation. The marker set identified on CD4^+^ Vδ1^+^ γδT-cell clones characterizes different progenitors, namely lin^−^ multipotent hematopoietic progenitors (MPP) as well as CLP in human bone marrow, as well as linlo ETPs, and canonical DN1 in the thymus (1). Like DN1-stage T-cell progenitors, CD4^+^ Vδ1^+^ γδT-cell clones were CD34^+^ CD38^+^ CD1a^−^ (Figure 1D).

**Figure 2:**
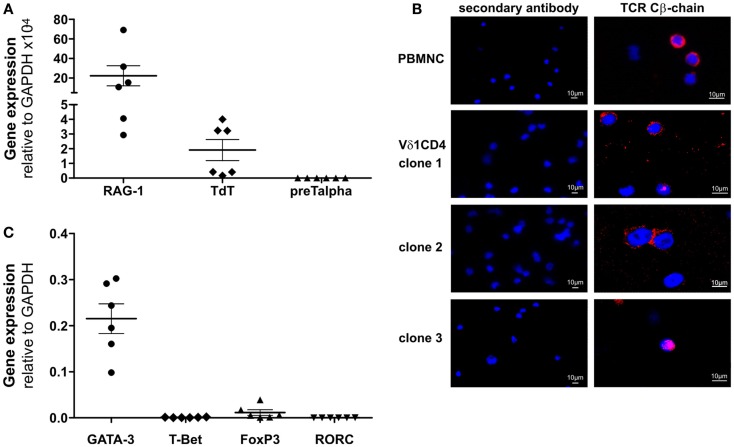
**CD4^+^ Vδ1^+^ T-cell clones express RAG and TdT, carry TCR-β chain in their cytoplasm and transcribe GATA-3. (A)** CD4^+^ Vδ1^+^ T-cell clones express RAG mRNA (mean: 23.36 ± 10.27 SEM), TdT mRNA (mean: 1.91 ± 0.72 SEM) but lack pre-Tα mRNA. Shown are mean ± SEM; **(B)** Vβ-chain protein (red) in the cytoplasm of CD4^+^ Vδ1^+^ clones grown under standard culture conditions, which mimic mild inflammation [blue: DAPI; Axiophot microscope (Zeiss)]. **(C)** Transcription factors expressed by CD4^+^ Vδ1^+^ T-cell clones, determined by real-time RT-PCR. GATA-3 (mean: 0.22 ± 0.03 SEM), T-bet (mean: 0.0009 ± 0.0002 SEM), Foxp3 (mean: 0.01 ± 0.006 SEM), and RORC (mean: 0.0001 ± 0.00008 SEM).

**Figure 3:**
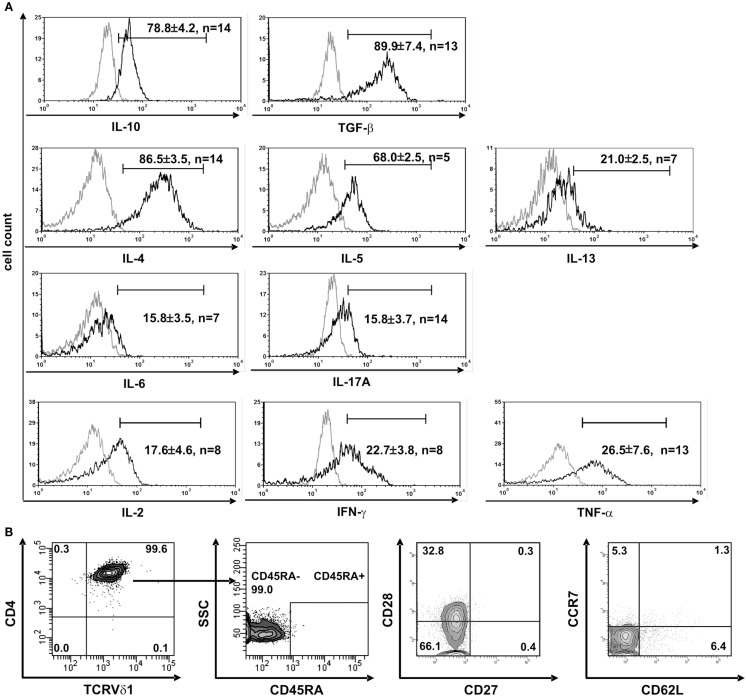
**CD4^+^ Vδ1^+^ T-cell clones have a T_H_0-cytokine profile and are effector-memory cells**. **(A)** CD4^+^ Vδ1^+^ clones produce regulatory IL-10 and TGF-β, T_H_2 cytokines IL-4, -5, and -13, and to a lesser extent proinflammatory IL-17A and IL-6, and the T_H_1-cytokines IL-2, IFN-γ, and TNF-α after stimulation with PMA/ionomycin. **(B)** CD4^+^ Vδ1^+^ clones were not naïve but belonged to the effector memory; they were CD45RA^−^, CD27^−^, CCR7^−^, and predominantly CD62L^−^ and CD28^±^.

Clones that were established directly from the bone marrow – the place where hematopoietic stem and progenitor cells reside – expressed significantly higher quantities of CD135 (*p* = 0.0182) (69.5 ± 3.6% cells positive/clone, *n* = 4) than peripheral blood-derived clones did (48.7 ± 6.8% cells positive/clone, *n* = 4) (Figure S1C in Supplementary Material), which is evidence for the presence of a more primitive precursor type in the bone marrow. Although CD4^+^ Vδ1^+^ γδT-cell clones did not initially express CD2 on their surface, they did so rapidly in the course of cultivation. This is additional evidence of the CD4^+^ Vδ1^+^ T-cell clones’ premature phenotype (Figure 1D). Moreover, CD4^+^ Vδ1^+^ clones transcribed RAG and TdT (Figure 2A), had fully rearranged TCR-β loci (Figure S1D in Supplementary Material), and the TCR Vβ protein was readily detectable in the cytoplasm (Figure 2B) and on the cell surface (Figure S2A in Supplementary Material). Thus established CD4^+^ Vδ1^+^ clones were Vδ1^+^Cβ^+^ but not TCRαβ^+^. CD4^+^ Vδ1^+^ clones were negative for pre-Tα (*n* = 9) (Figure 2A). In newly established clones, fully rearranged Vα segments were found in rare cases in periphery-derived clones, though never in LPH-derived CD4^+^ Vδ1^+^ clones (not shown). This suggests that the precursors found in the bone marrow are more primitive. GATA-3 was the major transcription factor while T-bet, RORc, and Foxp3 were only transcribed at very low levels (Figure 2C). CD4^+^ Vδ1^+^ γδT-cell clones spontaneously produced low level regulatory, T_H_1- and T_H_2-related, and proinflammatory cytokines (TGF-β, IL-2, -4, -5, -6, -10, -13, -17A, IFN-γ, and TNF-α) in standard culture conditions after stimulating the cells with PMA/ionomycin (Figure 3A). The CD4^+^ Vδ1^+^ γδT-cell clones did not express the CD45RA antigen, which clearly distinguishes them from recent thymic emigrants (Figure 3B). CD4^+^ Vδ1^+^ γδT-cell clones were CD45RO^+^, CD45RA^−^, CD62L^−^, CD27^−^, and CCR7^−^, and can thus be classified as effector-memory cells (Figure 3B).

Thus, CD4^+^ Vδ1^+^ γδT-cell clones expressed marker molecules that are characteristic for a thymus-seeding progenitor (TSP), as well as DN1–DN4 stage thymocytes and an ISP thymocyte, which in humans is CD4^+^.

### CD4^+^ Vδ1^+^ γδT-cell clones express chemokine receptors that direct circulating T cells to sites of inflammation

**Figure 4:**
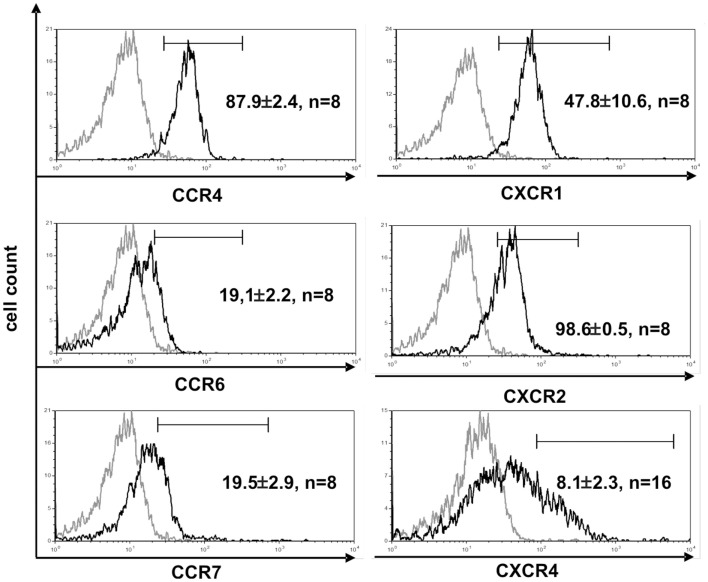
**CD4^+^ Vδ1^+^ T-cell clones express chemokine receptors associated with inflammation**. Vδ1^+^ CD4^+^ clones show strong expression of CCR4, CXCR1, and -2, and express CCR6^lo^, CCR7^lo^, and CXCR4^lo^. Histogram marker shows cells that are positive for the antigen of interest. Numbers indicate mean ± SEM of CD4^+^ Vδ1^+^ T cells that stain positive for the respective marker (given in %). Each histogram shows one representative experiment of all clones tested. Numbers of clones tested are given in each histogram. Gray line: isotype control.

The function of T cells is strongly connected with their microenvironment. CD4^+^ Vδ1^+^ γδT cells did not express the chemokine receptors CCR9 (8/9) and CCR7 (9/9), which makes homing to the thymus less likely (Figure 4) (45). However, they expressed five chemokine receptors: CCR4 (46, 47), CXCR1/CXCR2 (48), CCR6 (49), and CXCR4 (50), which direct the movement of circulating T cells to sites of tissue injury, infection, inflammation, and under physiological conditions to the skin (46, 47) (Figure 4).

### CD4^+^ Vδ1^+^ γδT-cell clones can change their TCR into αβTCR and mature into functional αβT cells

**Figure 5:**
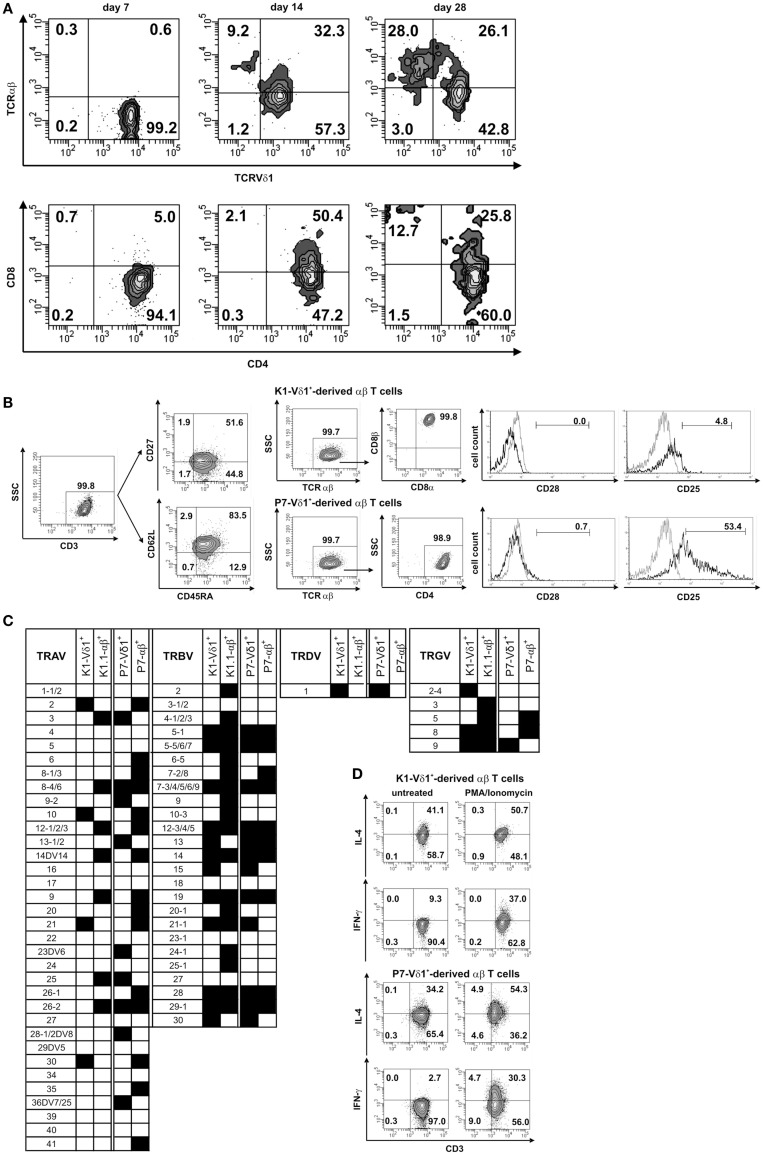
**CD4^+^ Vδ1^+^ T-cell clones change their TCR into TCR-αβ and can change their co-receptor**. Phenotypic, molecular, and functional characterization of αβ T cell lines exemplarily shown for lines derived from two different clones. **(A)** Process of transdifferentiation in a CD4^+^ Vδ1^+^ clone. Change of TCR expression (top), and change of co-receptor expression (bottom). **(B)** Resulting αβT-cell populations were heterogeneous. Exemplarily shown are two T-cell lines derived from two different clones. Their T-cell lines were terminally differentiated CD45RA-re-expressing T effector-memory cells (TEMRA). Clone K1-Vδ1^+^-derived αβ T cells were CD8αβ^+^ and did not express CD28 and CD25. Clone P7-Vδ1^+^derived αβT cells were CD4^+^, lacked CD28 but expressed CD25^int^. **(C)** Molecular analysis of TCR chain δ-, γ-, β-, and α-transcripts in two CD4^+^ Vδ1^+^ clones (K1-Vδ1^+^ and P7-Vδ1^+^) and two of the derived αβ T-cell lines (K1.1-αβ^+^ and P7-αβ^+^). The generated αβT-cell lines transcribe γ chain(s), but no δ chain. **(D)** Clone K1- and P7-Vδ1^+^-derived αβT cells constitutively secrete IL-4, but not IFN-γ; upon exposure to PMA/ionomycin they increase IL-4 secretion and secrete IFN-γ *de novo*. Gray line: isotype control.

CD4^+^ Vδ1^+^ γδT-cell clones turned out to be extremely long-lived. When grown under standard culture conditions, the CD4^+^ Vδ1^+^ γδT-cell clones successively downregulated the expression of CD34 on the cell surface (not shown) and some expressed αβ instead of γδTCR (Figure 5A, top). TCR change occurred rarely under standard culture conditions – only 1 out of 50 clones changed their Vδ1^+^ TCR into αβTCR. TCR change occurred either within days or up to 5 months after the establishment of the cell culture [Vδ1^+^ clone K1: +3 months and +5 months (different culture plate), Vδ1^+^ clone P7: +3 months] (Figure 5A, top). The cells remained either SP CD4^+^ or changed their co-receptor from SP CD4^+^ to DP CD4^+^/CD8^dim^. This led to pools of cells that were either SP CD4^+^ and/or SP CD8^+^ (Figure 5A, bottom). The CD4^+^Vδ1^+^ T-cell clone-derived TCRαβ^+^ T-cell line K1.1 (K1.1-αβ^+^) almost exclusively expressed surface TRBV11-2^+^ and -4.1^+^, and T-cell line K1.2 (K1.2-αβ^+^) almost exclusively TRBV27^+^ and -19^+^ (Figures S2B,C in Supplementary Material). The clone P7-derived TCRαβ^+^ T-cell line did not express TCR Vβ families that are detectable with available TCR antibodies (data not shown). All CD4^+^ Vδ1^+^ T-cell clone-derived αβT cells were T10B9.1A-31^+^ (framework epitope of αβTCR heterodimer), WT31^+^ (CD3ε), CD3^+^, Vδ1neg, γδTCRneg, CD25lo/neg, CD28neg, and were also terminally differentiated effector-memory RA-re-expressing T cells (TEMRA) (Figure 5B). The CD4^+^ Vδ1^+^ T-cell clone-derived αβT-cell lines lacked CD103, and therefore did not have a tissue-resident memory (TRM) phenotype (not shown). Furthermore, they did not transcribe TCR-δ segments, which is due to the deletion of the TCR-δ locus as a result of the recombination of the genes that encode the TCR-α chain. Nevertheless, the αβT-cell lines still transcribed rearranged γ chain segments (Figure 5C). Under standard culture conditions, the αβT cells derived from clone K1-Vδ1^+^ and P7-Vδ1^+^ produced IL-4 but not IFN-γ. In addition, they responded to (PMA)/ionomycin stimulation with a (subtle) increase in IL-4 release and with the substantial *de novo* production of IFN-γ, demonstrating their functionality (Figure 5D). The αβT cells responded poorly to mitogenic stimuli (data not shown). The clone C3-23-derived αβT-cell line produced IFN-γ (41% of the cells) and IL-10 (55% of the cells) when stimulated with PMA/ionomycin. These are the same cytokines as those produced in lower quantities under standard culture conditions (not shown). αβT-cell lines derived from other clones produced mainly IFN-γ and IL-10.

### Developmental steps of CD4^+^ Vδ1^+^ clone cells in the process of transdifferentiation

**Figure 6:**
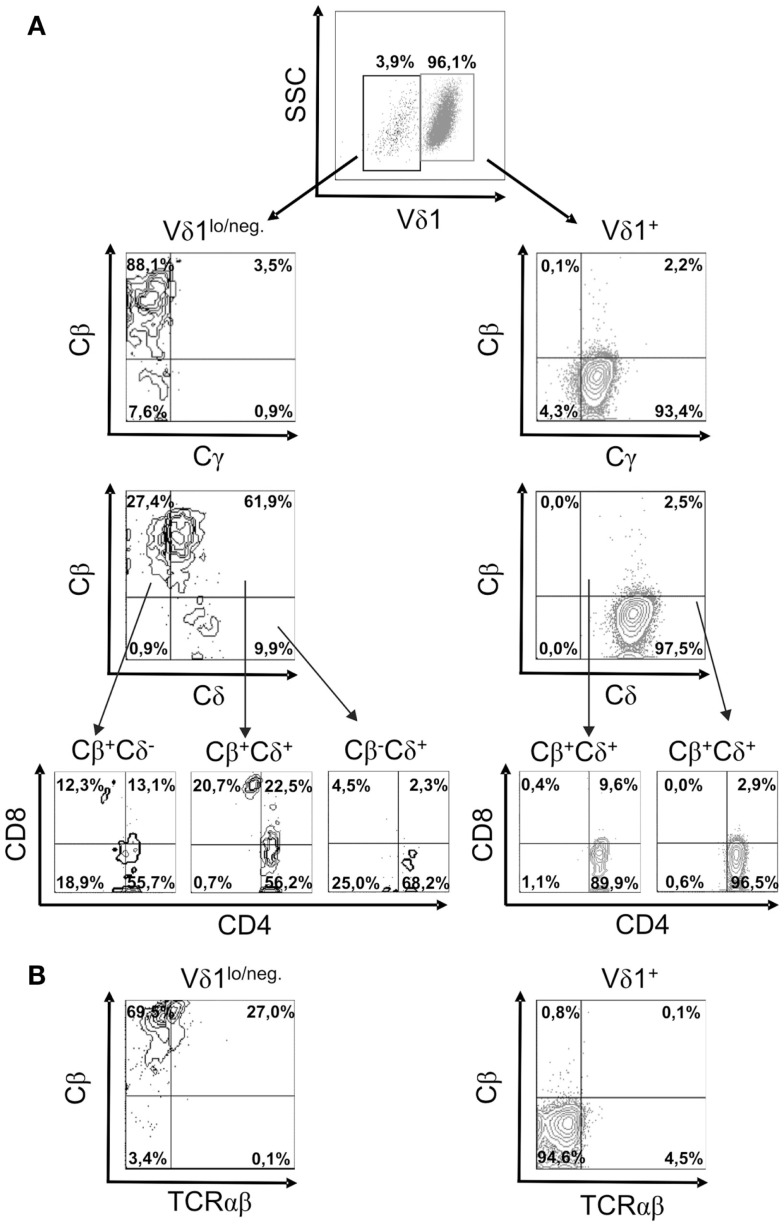
**Characterization of the subsets of a CD4^+^ Vδ1^+^ clone cells that changes TCR-γδ into TCR-αβ**. **(A)** Vδ1^+^ and Vδ1^lo/neg.^ subpopulation of CD4^+^ Vδ1^+^ T-cell clones examined for the expression of Cγ, Cδ, Cβ, and Cα (as a TCR-αβ framework epitope) as well as co-receptors. A clone representative of seven analyzed clones is shown. **(B)** TCR-αβ^+^ cells in Vδ1^hi^ and Vδ1^lo/neg.^ Cβ^+^-expressing Vδ1^+^ CD4^+^ T-cell clone fractions. A clone representative of seven analyzed clones is shown.

In order to study TCR re-organization during transdifferentiation, the expression of the constant region of the TCR chains, Cδ, Cγ, Cβ (BMA031) and Cα (as a TCR-αβ-associated framework epitope), in CD4^+^ Vδ1^+^ clones undergoing TCR change was monitored. CD4^+^ Vδ1^+^ T cells consistently downregulated the expression of the TCR-γ and TCR-δ chains, thus becoming Vδ1^dim^/Cγ^dim^, and then Vδ1^lo/neg.^ cells that lacked Cγ (Figure 6A). Vδ1^lo/neg.^Cγ^neg.^ cells expressed Cβ on their cell surface. These Vδ1^lo/neg.^Cβ -expressing cells were CD4^+^CD8^+^ DP and eventually developed into Vδ1^neg.^Cβ^+^ SP CD4^+^ or SP CD8^+^ T cells (Figure 6A). In order to identify the number of αβT cells in the cultures, we stained for Cβ and TCRαβ in Vδ1^+^ cell cultures and found that 84.6% ± 8.0 SD of all Vδ1^lo^/^neg.^ cells expressed Cβ, while 26.6% ± 6.64 SD) of all Cβ^+^ Vδ1^lo^/^neg.^ cells stained positive for TCRαβ (Figure 6B). Cβ^+^ cells were CD4^+^CD8^+^ DP (29.75% ± 15 SD), or CD8^+^ (8.37% ± 4.62 SD), or CD4^+^ (58.38% ± 11.21 SD), and in rare cases DN (3.5% ± 5.4 SD) (an example of which is shown for one clone in Figure 6A). TCRαβ^lo^ cells then upregulated αβTCR and could not be distinguished from peripheral αβT cells (not shown).

### Inflammation triggers differentiation of Vδ1^+^ γδT cells into αβT cells

As TCR change occurred in only one out of 50 established CD4^+^ Vδ1^+^ γδT-cell clones, we purified Vδ1+ γδT cells, including the DN, CD8^+^, and CD4^+^ subsets from peripheral blood mononuclear cells (PBMNCs) of healthy human donors. These cells (panVδ1^+^) were subsequently used to study whether inflammatory stimuli trigger transdifferentiation. For that two different inflammatory settings were compared: standard culture conditions were designated as mild inflammation, whereas stronger inflammatory stimuli were termed overt inflammation.

**Figure 7:**
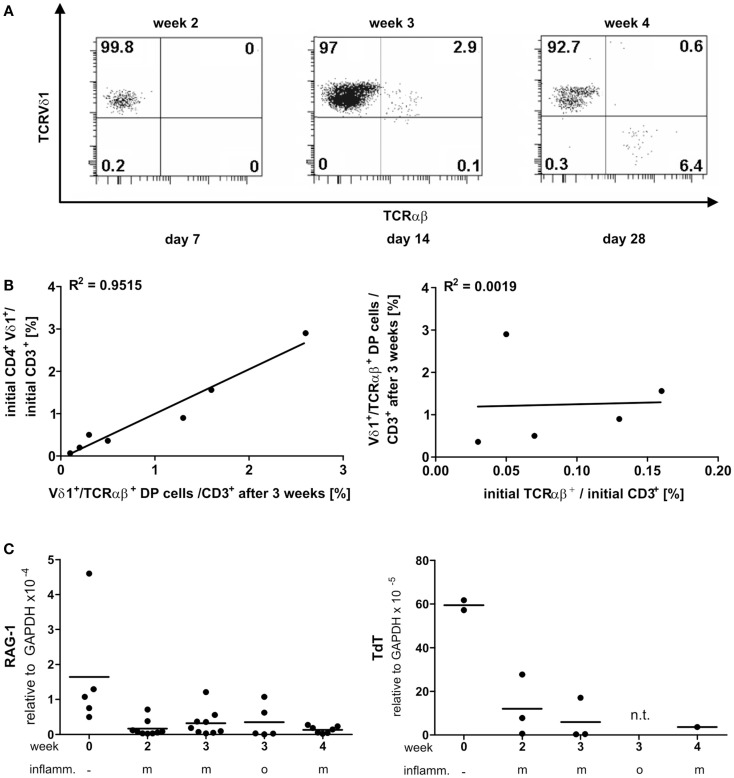
**Vδ1^+^ T cells differentiate into αβT cells**. RAG and TdT expression in panVδ1^+^ cultures, native and over time in different inflammatory culture conditions; correlation of TCR DP cells with CD4^+^Vδ1^+^ and residual αβT-cell numbers in initial panVδ1^+^ T-cell pools. **(A)** panVδ1^+^ T cells cultivated in the presence of IL-7, PHA, IL-2, and irradiated allogeneic feeder cells gave rise to a cell fraction that co-expressed Vδ1 and TCR-αβ after 3 weeks, before differentiating into TCR-αβ^+^ T cells. Series of dot plots are representative of 12 independent experiments. **(B)** Number of Vδ1^+^/TCR-αβ^+^ cells varied between individuals (mean: 0.926%/Vδ1^+^; range 0.1–3%) (right) as did the number of produced αβT cells (mean: 1.82%/Vδ1^+^; range 0.2–6.0%) (left); Vδ1^+^/TCR-αβ^+^ double-positive cells in week 3 of culture corresponded with the number of CD4^+^ Vδ1^+^ T cells in initial Vδ1^+^ T-cell pool (left). There was no correlation between residual TCR-αβ^+^ T cells after Vδ1^+^ cell separation and set-up of initial Vδ1^+^ T-cell culture and the number of Vδ1^+^/TCR-αβ^+^ double-positive T cells after 3 weeks of culture (right). Each dot represents one independent experiment. **(C)** After initial depression, RAG-1 and TdT quantities subtly increased in Vδ1^+^ cell cultures and were detectable simultaneously with the appearance of Vδ1^+^/TCR-αβ^+^ coexpressing cells. m, Mild inflammation (IL-7, PHA, IL-2, and irradiated allogeneic feeder cells); o, overt inflammation (exposure to strong inflammatory stimuli in week 3, conditions described in the Section “Materials and Methods”); week 0, freshly isolated, native Vδ1^+^ cells were analyzed.

**Figure 8:**
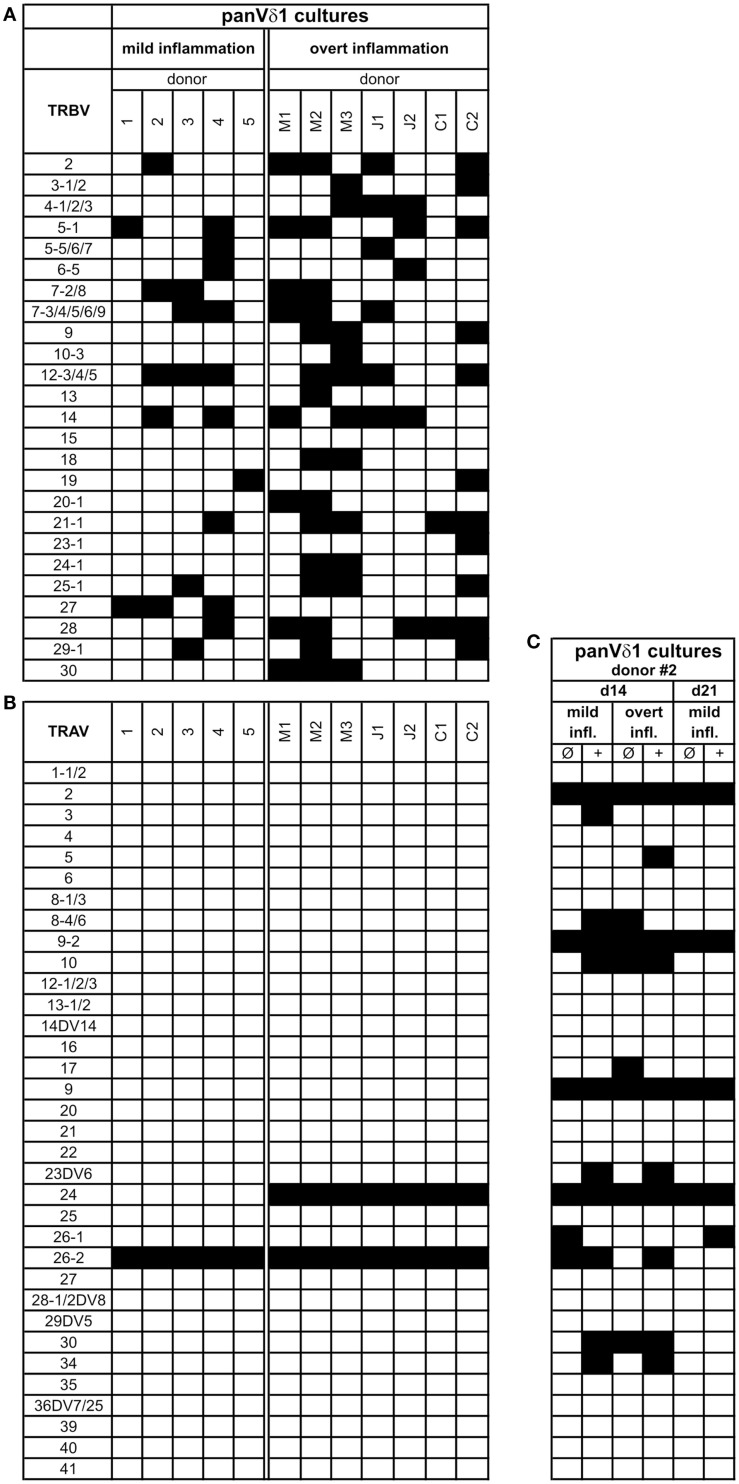
**Repertoire of fully rearranged and transcribed TRBV and TRAV segments in panVδ1^+^ T cell pools that were cultivated in different inflammatory environments**. **(A)** TRBV-segment usage and **(B)** TRAV-segment usage after 3 weeks of culture **(C)** Comparison of Vα-segment usage of panVδ1^+^ T cells from one donor (#2) in different modified inflammatory environments. +, Treatment combined with anti-CD40; IL-15 (10 ng/mL) was added to all cultures. Black squares indicate expression of transcripts of rearranged TCR chains.

PanVδ1^+^ T cells cultivated in the mild inflammatory environment (standard culture, see Materials and Methods) gave rise to a subset of T cells that transdifferentiated into TCRαβ^+^ T cells (*n* = 12) (Figure 7A) within 3–4 weeks. Vδ1^+^ cells sequentially changed their TCR, reorganized their Vδ1^+^ γδTCRint/lo phenotype to phenotype Vδ1int/lo/TCR-αβlo, and from the latter to phenotype Vδ1^−^γδ^−^TCR-αβ^+^ (Figure 7A). The percentage of Vδ1int/lo/TCR-αβlo DP cells correlated exactly with the percentage of CD4^+^ cells in the initial panVδ1^+^ T-cell pools. However, the number of Vδ1int/lo/TCR-αβlo DP cells did not correlate with the low number of αβT cell contaminants found in the initial culture pool (Figure 7B). The number of CD4^+^ Vδ1^+^ T cells within the panVδ1^+^ T-cell pool varied greatly between individuals (mean: 0.926% of all Vδ1^+^ T cells; range: 0.1–3.0%), as did the number of αβT cells generated from panVδ1^+^ cell pools (mean 1.82% of input Vδ1^+^ T cells; range 0.2–6.4%) (Figure 7B). Concomitantly with TCR change, RAG-1 and TdT mRNA was detected in panVδ1^+^ T-cell pools. TdT is positively regulated by Tβ4 of which large amounts are expressed by epithelial (Vδ1^+^) γδT cells (51) (Figure 7C). Additionally, mRNA isolated from the panVδ1^+^ cultures showed that functionally rearranged TRBV and TRAV segments emerged at the same time as Vδ1int/lo/TCR-αβlo intermediates (*n* = 5) did (Figures 8A,B). The modulation of inflammatory culture conditions resulted in similar, overlapping TRAV expression patterns (Figure 8C). In contrast, TCR-α chain transcripts were not present in the aliquots of the initial panVδ1^+^ T-cell pools. They were not present in panVδ1^+^ and pan γδT-cell pools of peripheral blood of healthy donors either (not shown). The transcription rate of pre-Tα was the same as in the controls with no template.

In the overt inflammatory approach panVδ1^+^ T cells received a combination of cytokines that are pivotal in acute and chronic inflammation, and monocytes, which had been preactivated with the same cocktail for three days (see Materials and Methods: overt inflammation).

**Figure 9:**
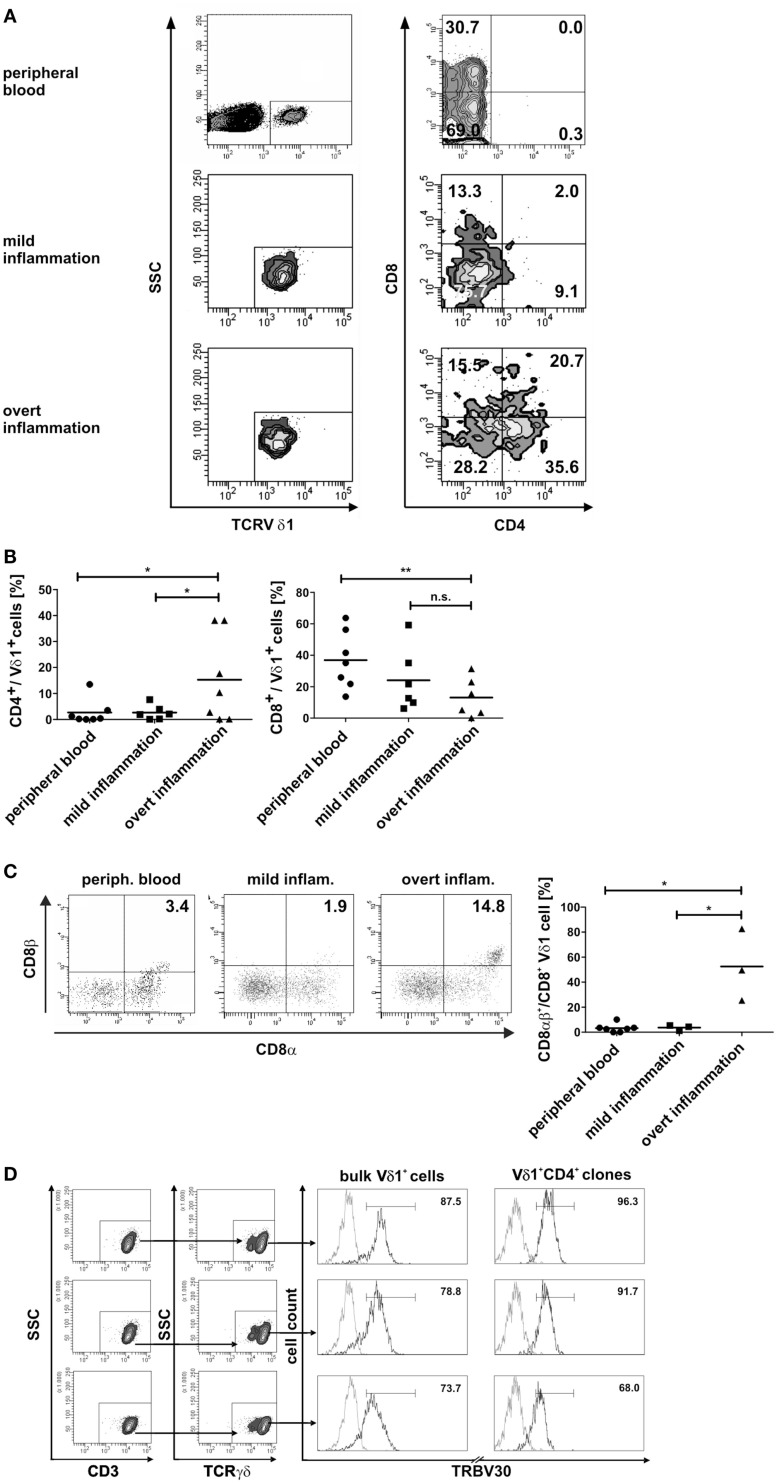
**Phenotypic changes of panVδ1^+^ T cells in inflammatory conditions**. **(A)** Inflammation-induced phenotype of DP CD4^+^ CD8^+^ Vδ1^+^ γδT cells. **(B)** *Ex vivo* overt inflammatory conditions significantly increased the number of CD4^+^ T cells in panVδ1^+^ cell cultures to an extent that was much higher than that seen in peripheral blood of healthy individuals (*p* = 0.0493) or in mild inflammation *in vitro* (*p* = 0.0487). In comparison to peripheral blood of healthy individuals, overt inflammation led to a significant reduction of the number of CD8^+^ Vδ1^+^ cells (*p* = 0.0097). **(C)** Overt inflammation induced the expression of the CD8αβ heterodimer in a significant proportion of CD8^+^ Vδ1^+^ cells (*p* = 0.0452) in 3/3 donors tested. **(D)** panVδ1^+^ cells show TRBV30 chains on the cell surface after 7 days of (both: mild and overt) inflammation (5/5 tested, shown are 3/5); TRBV30 was also observed on clones that were chronically exposed to mild inflammation (7/7 tested, 3/7 are shown). Gray line: isotype control. Histogram marker shows range of cells that stained positive for the antigen under investigation. Numbers indicate mean ± SEM of Vδ1^+^ (CD4^+^) T cells that stained positive for the respective marker (given in %). The histograms show three representative experiments for panVδ1^+^ T cells and CD4^+^ Vδ1^+^ clones stained with TRBV30. TRBV30 expression was determined in panVδ1^+^ cultures with 4 different donors in 3 independent experiments each, and in 7 CD4^+^ Vδ1^+^ clones.

Fundamental phenotypic changes were observed at the protein and transcriptional level. A significant upregulation of CD4 (4.3-fold; *p* = 0.0487) created (more) Vδ1^+^ SP CD4^+^ cells and the novel phenotype of Vδ1^+^ CD4^+^ CD8^+^ DP T cells in five out of seven cultures (Figures 9A,B). Simultaneously, the CD8 co-receptor was downmodulated (Figure 9B) and the CD8αα homodimer was replaced by the CD8αβ heterodimer in a significant proportion of CD8^+^ Vδ1^+^ T cells (Figure 9C) (*p* = 0.0452, *n* = 6). Interestingly, panVδ1^+^ T cells became weakly positive for Vβ chains on their surface (Vβ^+^); most of them expressed TRBV30 (*n* = 12) as shown by FACS analysis (Figure 9D). In addition, other Vδ1^+^ T cells in the panVδ1 T cell pool carried a varying number of individual TRBV chains on their surface (not shown). It is worth noting that strong inflammation (as indicated in the Section “Materials and Methods”) was not associated with the generation of DP Vδ1^+^ /TCR-αβ^+^ or αβT cells. Strong inflammation did not affect the transcription of RAG (Figure 7C), Vβ-segment usage (Figure 8A) and complexity score (native: 20.00 ± 7.21 SEM, mild infl.: 7.67 ± 2.96 SEM; overt infl.: 28.43 ± 7.38 SEM). However, emergence of full-length transcripts of in-frame TRAV-26-2 and TRAV24 rearrangements in cultures of all donors demonstrated that the TCR-α locus had been accessible and was recombined (*n* = 7) (Figure 8B). TdT was also induced, evidenced through long stretches of *N* nucleotides in the TRAV24 sequences (Table 1). Vα rearrangements, both in panVδ1^+^ and CD4^+^ Vδ1^+^ clone cultures, exactly followed the hierarchical order of the thymic rearrangement process that reconciles the sequential opening of the 3′ end of the V region and the 5′ end of the J region (52).

**Table 1:** **CDR3 region peptide composition of rearranged TRAV segments in panVδ1^+^ T cells induced by mild and overt inflammation and CDR3 regions of rearranged TRBV and TRAV segments of αβT cells derived from CD4^+^ Vδ1^+^ clone K1-Vδ1^+^ (K1.1-αβ^+^) under mild inflammatory conditions**.

Donor	No. of exp.	TRAV	TRAJ	3′of V-segment	CDR3	J segment	Public motif type	Reference
**MILD INFLAMMATION**								
1	1	26-2	41	CI	LRGL	NSGYALNFCKRT…	2	N/A
	1	26-2	41	CI	LRGS	NSGYALNFCKRT…	2	N/A
2	1	26-2	41	CI	LRDW	NSGYALNFGKGT…	3	N/A
3	1	26-2	41	CI	LRDW	NSGYALNFGKGT…	3	N/A
4	1	26-2	41	CI	LRDL	NSGYALLFCKRT…	3	N/A
5	1	26-2	41	CI	LRDL	NSGYALLFCKRT…	3	N/A
**OVERT INFLAMMATION**								
1	1	26-2	41	CI	LRGL	NSGYALNFGKGT…	3	N/A
2	1	26-2	41	CI	LRDL	NSGYALNFGKGT…	3	N/A
3	1	26-2	41	CI	LRDL	NSGYALNFGKGT…	3	N/A
4	2	26-2	41	CI	LRDL	NSGYALNFGKGT…	3	N/A
5	1	26-2	41	CI	LRDL	NSGYALNFGKGT…	3	N/A
6	3	26-2	41	CI	LRGL	NSGYALNFGKGT…	3	N/A
7	1	26-2	41	CI	LRDL	NSGYALNFGKGT…	3	N/A
4	3	24	3	LA	RRNDG	SASKIIFGSGT…	3	N/A
5	2	24	3	LA	RRNDG	SASKIIFGSGT…	3	N/A
6	2	24	3	LA	RRNDG	SASKIIFGSGT…	3	N/A
**α CHAINS OF αβT CELLS DERIVED FROM CLONE K1 UNDER MILD INFLAMMATORY CONDITIONS**								
K1.1-αβ^+^		3	5	CA	VRD	DTGRRALTFGSGT	–	N/A
		14DV4	17	CA	MREGRF	KAAGNKLTFGGGT…	2	(53)
		25	10	CA	G	TGGGNKLTFGTGT…	–	N/A
		26-2	52	CQ	LSTARTPTP	SQDAVVHSSISQGST	1	(54, 55)
**β CHAINS OF αβT CELLS DERIVED FROM CLONE K1 UNDER MILD INFLAMMATORY CONDITIONS**								
K1.1-αβ^+^		11-2	1-1	CAS	RRG	TEAFFGQGT…	2	(56), (57)
		19	2-7	CASS	SNGQGV	YEQYFGPGT…	1	(58)
		27	2-3	CASS	LGDRVA	DTQYFGPGT…	2	(59)

*CDR3 regions of rearranged TRAV segments in panVδ1^+^ cultures and of rearranged TRBV and TRAV segments in K1-derived αβT cells. (A) top: mild inflammation induced exclusively TRAV-26-2JC transcripts in panVδ1^+^ T cells, transcripts of donors J, K, and P were sequenced; overt inflammation also induced TRAV24; TRAV-derived transcript sequence analysis is shown for all donors and all experiments. (B) CDR3 sequences of TRBV and TRAV segments, detected in spectratype analysis as single peaks, were determined from K1-derived αβT cells (K1-17): 3/18 single peaks of TRBV-segments, 4/4 single peaks of TRAV segments. Sequences were derived from cDNA transcripts after PCR-based spectratype analysis*.

### Identification of the Vδ1^+^CD34^dim^ precursor *in vivo* and its transdifferentiation intermediates in physiological and inflamed tissue

**Figure 10:**
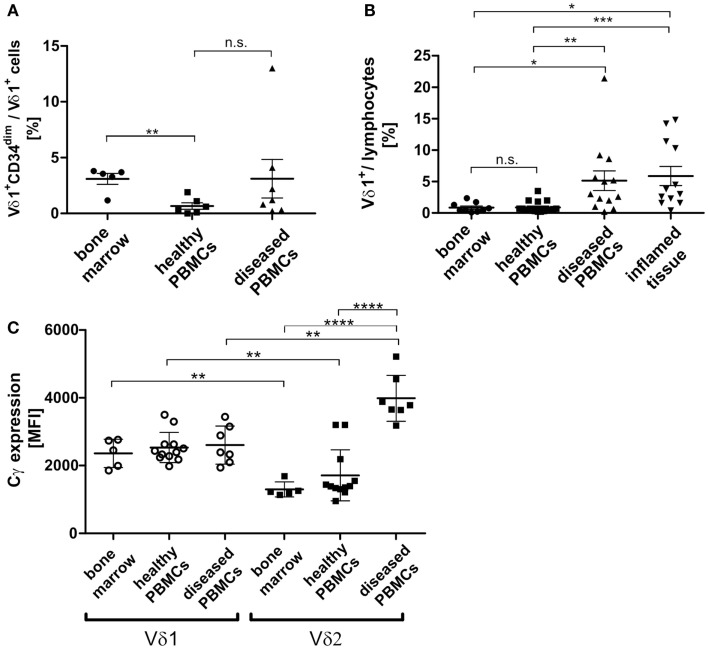
**Vδ1^+^ lymphocytes and Vδ1^+^ CD34^dim^ T cell population in blood and marrow**. **(A)** Percentage of Vδ1^+^ CD34^dim^ T cells/Vδ1^+^ lymphocytes in peripheral blood of healthy (*n* = 6) and diseased donors (*n* = 7) and healthy bone marrow (*n* = 5). The percentage of Vδ1^+^ CD34^dim^ T cells/Vδ1^+^ lymphocytes was significantly higher in native bone marrow compared to peripheral blood from healthy donors (*p* = 0.0017). **(B)** The quantity of Vδ1^+^ lymphocytes was (highly) significantly increased in the peripheral blood of diseased (*n* = 13; mean: 5.13 ± 1.57 SEM; *p* = 0.0096) and inflamed tissue (*n* = 12; mean: 5.88 ± 1.52 SEM; *p* = 0.0388) compared to the quantity observed in the blood of healthy individuals (*n* = 17; mean: 0.89 ± 0.22 SEM). Vδ1^+^ lymphocytes were significantly more frequent in peripheral blood (*p* = 0.0367) and body fluids (*p* = 0.0107) of diseased individuals than in bone marrow (*n* = 9 mean: 0.85 ± 0.26 SEM). **(C)** Absolute expression level of TCR-γ chain on Vδ1^+^ and Vδ2^+^ cells in healthy (*n* = 12) and diseased (*n* = 7) peripheral blood and bone marrow (*n* = 5). Blood samples included blood from patients with uncharacterized viral infection (*n* = 3), pneumonia (*n* = 1), cystitis (*n* = 2), nephritis (*n* = 1) (Cγ MFI mean: Vδ1^bone marrow^ 2360 ± 188 SEM versus Vδ2^bone marrow^ 1298 ± 99 SEM; Vδ1^healthy^ 2533 ± 129 SEM versus Vδ2^healthy^ 1711 ± 218SEM; Vδ1^diseased^ 2606 ± 213SEM versus Vδ2 ^diseased^ 3986 ± 256 SEM).

In order to study the significance of the Vδ1^+^CD34^dim^ precursor and its transdifferentiation *in vivo*, its frequency in the bone marrow was determined. Of all lymphocytes in the bone marrow, 0.039% were Vδ1^+^CD34^dim^ precursors (*n* = 8, not shown), which correlated with the number of Vδ1^+^CD34^dim^ T cells per Vδ1^+^ subset in the peripheral blood of diseased individuals, but was significantly different from their numbers in healthy PBMNCs (Figure 10A). However, the overall number of Vδ1^+^ T cells was significantly higher in the peripheral blood of diseased subjects [4.6-fold; range: healthy 0.3–2.0 (*n* = 6), diseased 0.6–8.6 (*n* = 7)] (Figure 10B), which influences the absolute number of CD34^dim^ cells in the periphery (Figure 10A). Our diseased cohort included individuals that suffered from viral infections (*n* = 4), lupus erythematosus (*n* = 1), vitiligo (*n* = 1), and a viral infection associated with chronic fatigue syndrome (*n* = 1). The Vδ1^+^CD34^dim^ precursors in the peripheral blood were mostly DN (52.08%) and CD8 (42.66%) (not shown), which is consistent with the finding that CD4^+^ is upregulated by inflammatory stimuli (Figure 9A).

### TCR-γ chain expression in Vδ1^+^ T cells *in vivo*

As TCR change occurred in TCR-γ^low/neg.^-expressing cells (Cγ^lo/neg.^), we determined TCR-γ expression *in vivo* (freshly drawn peripheral blood) and in native healthy bone marrow. Unexpectedly, TCR-γ chain (Cγ) expression was significantly higher in Vδ1^+^ than in Vδ2^+^ cells in the blood of healthy donors and in bone marrow. In contrast, there was a significantly higher expression of Cγ in the Vδ2^+^ than in the Vδ1^+^ cells in the blood of diseased volunteers and in bone marrow. When comparing absolute Cγ expression, the Vδ2^+^ subset showed a significant upregulation of TCR-γ chain expression during inflammation, whereas the Vδ1^+^ subset expressed the γ-chain constitutively in bone marrow and PBMNCs irrespective of inflammatory stimuli. Blood samples were taken from individuals suffering from uncharacterized viral infection (*n* = 3), pneumonia (*n* = 1), cystitis (*n* = 2), nephritis (*n* = 1); (Cγ MFI mean: Vδ1^bone marrow^ 2360 ± 188 SEM versus Vδ2^bone marrow^ 1298 ± 99 SEM; Vδ1^healthy^ 2533 ± 129 SEM versus Vδ2^healthy^ 1711 ± 218SEM; Vδ1^diseased^ 2606 ± 213SEM versus Vδ2 ^diseased^ 3986 ± 256 SEM) (Figure 10C).

### TCR-β chain expression in Vδ1^+^ T cells *in vivo*

**Figure 11:**
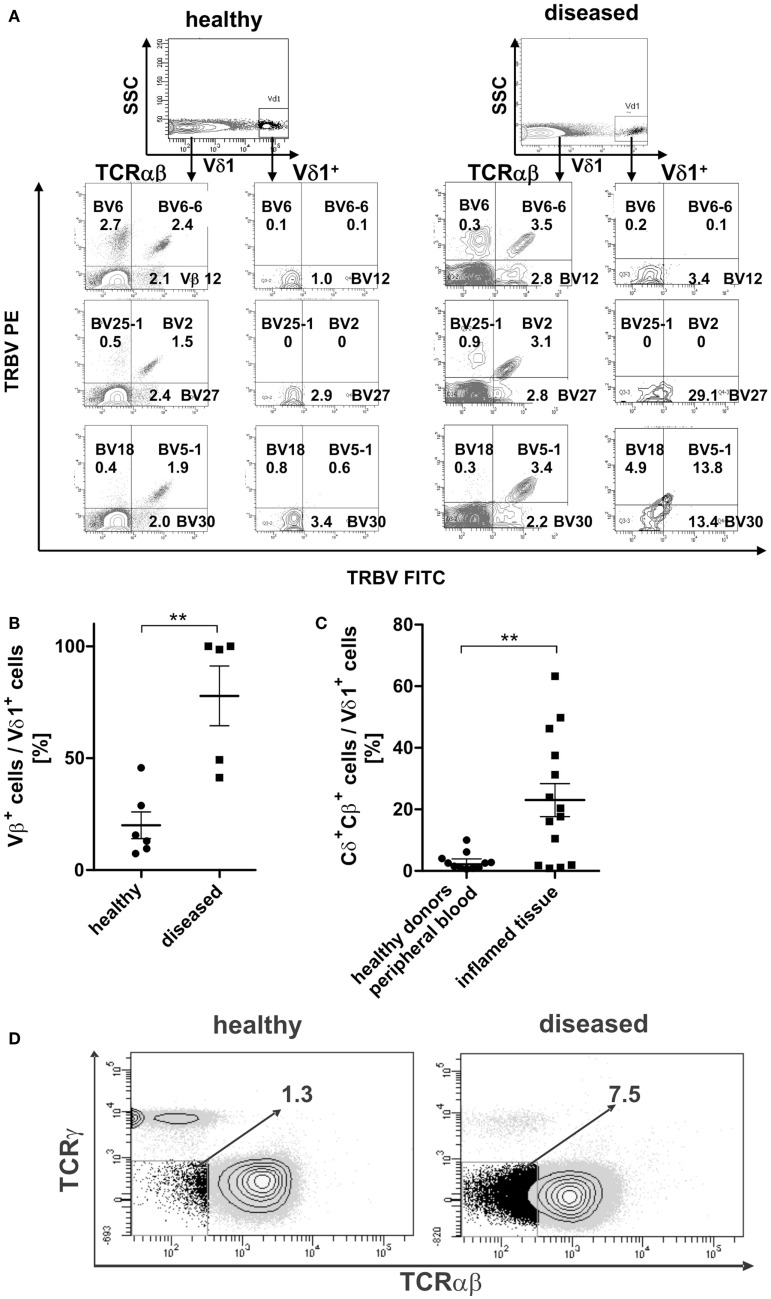
**Surface Vβ expression of αβT cells in comparison with peripheral Vδ1^+^ T cells in healthy and diseased individuals**. **(A)** Representative Vβ family staining pattern (exemplarily shown are nine different Vβ families) on αβT cells and Vδ1^+^ T cells in peripheral blood MNCs shown for one healthy individual and for one individual suffering from viral infection. MNCs were stained with the TCR Vβ Repertoire Kit (Beckman Coulter); three different Vβ family expressions are assessed in the same tube by combining three monoclonal antibodies (mAb) with only two fluorophores (see Materials and Methods for detailed information). **(B)** Percentage of Vβ^+^ expressing cells in the Vδ1 subset in healthy (*n* = 6) and diseased individuals (*n* = 5). **(C)** Percentage of Vδ1 cells with a Cδ^+^Cβ^+^ phenotype in healthy (*n* = 11) tissue differs significantly from that of body fluids of inflamed tissue (*n* = 14) (*p* = 0.0343). **(D)** CD3^+^ cells that are Cγ^neg.^ and TCR-αβ framework-epitope^neg.^ (DN for TCR) in healthy (*n* = 11) and diseased individuals (*n* = 4). One representative blot is shown.

TCR-β chain expression was determined for the Vδ1^+^ subset and the αβT-cell compartment in the peripheral blood of healthy and diseased individuals (Figure 11A). The percentage of Vβ^+^-expressing Vδ1^+^ cells differed highly significantly between healthy (*n* = 6; 20.0% ± 5.98 SEM) and diseased (*n* = 5; 77.8% ± 13.34 SEM) blood (Figure 11B).

### Vδ1^+^Cβ^+^ TCR intermediates in peripheral blood and at sites of inflammation

In order to assess whether the transdifferentiation of CD4^+^ Vδ1^+^ γδT cells is of physiological importance, we investigated body fluids of inflamed tissues for the presence of cellular intermediates of the transdifferentiational route. Vδ1^+^Cβ^+^ intermediates were present in significantly larger numbers in body fluids of inflamed tissue (synovia of arthritic joints, tumor-associated ascites, pleural effusion; mean expression 23% ± 5.38 SEM; range 0.8–63.2%) than in the peripheral blood of healthy individuals (mean expression healthy 3.0%; ±0.86 SEM; range 0.6–10.0%) (Figure 11C).

Given that the transdifferentiation intermediates express the δ1- and a β-chain for a δβ^+^ heterodimer, which must occur in the absence of the TCR-γ chain (60), and given that the α-chain is not yet rearranged and expressed, the expected resulting phenotype after FACS staining with the murine antibody that targets the γδTCR constant region (clone 11F2) and the αβTCR framework epitope (T10B9.1A-31) is CD3^+^, “TCRnegative,” CD4^+^ or CD8^+^. This phenotype was detected in all donors tested (Figure 11D).

**Figure 12:**
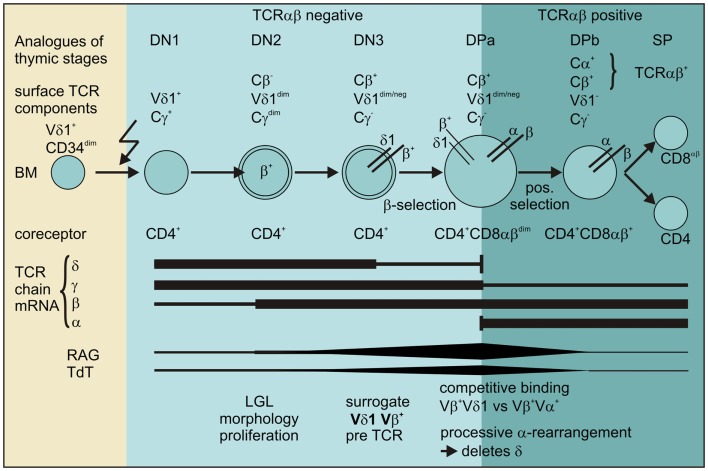
**Scheme for the transdifferentiation of Vδ1^+^ CD34^dim^ precursors into αβT cells inflammatory stimuli induce Vδ1^+^ CD34^dim^ precursors to move from the bone marrow into the periphery and express CD4^+^**. Chemokine receptors guide Vδ1^+^ CD4^+^ precursors to the sites of inflammation where inflammation-associated stimuli induce their development into αβT cells: Vδ1^+^ CD4^+^ cells sequentially rearrange, transcribe and express Vβ chain intracellularly, while downmodulating TCRγ and to a lesser extent Vδ1 expression. The Vβ chains then appear on the surface of Cγ^lo/neg.^ cells and δ^+^β^+^ precursors transit into the CD4^+^CD8^+^ DP stage where processive rearrangement in the α locus – accompanied by the expression of RAG and TdT – deletes the δ-chain, leading to the successive loss of Vδ1 on Vδ1^dim^Vβ^+^ cells. Vβ^+^ chains preferentially pair with newly formed α chains in Vδ1^dim/neg.^ Vβ^+^ cells that become weakly positive for TCR-αβ before they completely lose Vδ1 expression, upregulate TCR-αβ and become mature and functional SP CD4^+^ or CD8^+^ αβT cells.

Thus, our data provide evidence for the existence of a pathway for extrathymic αβT-cell development from the CD4^+^ Vδ1^+^ T-cell precursor as shown in Figure 12.

## Discussion

In this study, we identified the human Vδ1^+^ γδT-cell subset as a reservoir of a CD4^+^ cell entity that shows the cumulative expression of markers and molecules that are pivotal for T-cell progenitor phenotype and function namely CD34^dim^, FLT3^+^, c-kitlo, CD105^+^, and CXCR4^+^. Low level and simultaneous expression of cytokines characterizing multiple Th lineages conforms with the view that stem-cells possess a wide-open chromatin structure to maintain their multipotentiality, which is progressively lost during differentiation (61, 62). The combined expression of five different chemokine receptors that direct the movement of circulating T cells to sites of inflammation indicates a function of this progenitor in inflamed tissue. We provide phenotypic, transcriptional and functional evidence that initial CD34^dim^ CD4^+^ Vδ1^+^ γδT-cell clones can develop into functional αβT cells in *ex vivo* cultures. By monitoring surface expression of the constant region of the 4 different TCR chain loci γ, δ, β and α on CD34^dim^ CD4^+^ Vδ1^+^ γδT cell clones, we identified the CD4^+^ Vδ1^+^ γδT cells’ transdifferentiation into αβT cells as a sequential invariant process that is triggered by (mild) inflammatory stimuli (schematically shown in Figure 12). The re-organization of Vδ1^+^ TCR-γδ into TCR-αβ was associated with morphological and physiological changes of the CD4^+^ Vδ1^+^ γδT cells reproducing thymic T-cell development: cultured peripheral CD4^+^ Vδ1^+^ γδT cells increased in size, acquired an LGL-like morphology, downregulated TCR-γ and – to a lesser extent – TCR-δ; they proliferated vigorously, expressed functionally rearranged TCR-β chains on their surface, thus forming the Vδ1^+^Cβ^+^ cellular intermediates that traversed into the CD4^+^CD8^+^ DP stage. An increase in RAG and TdT activity preceded the induction of the rearrangement of the TCRα loci in exact thymic order before the Vδ1^+^Cβ^+^ intermediates shut down Vδ1 expression, expressed an αβTCR and developed into functional SP CD4^+^ or CD8^+^ αβT cells.

In order to exclude these observations as artifacts of *ex vivo* culture systems, we compared our findings with established literature data, and in order to identify its physiological relevance, we aimed to show analogs of this new developmental pathway *in vivo*. Several groups of researchers have shown that around 10–20% of all peripheral γδ T cells transcribe and express in-frame TCR-β rearrangements (63–65) that can guide αβT-cell development, resulting in fully functional, mature T cells (66). Consistent with these findings, CD4^+^ Vδ1^+^ clones transcribed in-frame TCR-β chain rearrangements, showed cytoplasmic protein expression of TCR-β chains, and expressed the TCR-β chain on their cell surface as they bound BMA031. This is in accordance with the findings of Miossec et al. (67) who showed that up to 45% of peripheral Vδ1^+^ cells bind Cβ region-specific monoclonal antibodies (mAb) BMA031. Using a set of mAbs that recognize the variable domain of 24 different human Vβ chains, we refined our analysis in order to demonstrate that peripheral Vδ1^+^ T cells of healthy individuals expressed the full spectrum of Vβ chains. While Miossec and other researchers showed that the Vδ1^+^ variable region can substitute Vα in functional T-cell receptor α-chains and thus serve as an agent for Vβ surface expression in the peripheral αβT cell subset (67–71), in the current study we identified a subset in every CD4^+^ Vδ1^+^ clone analyzed, which lacked surface Cα – because the clone culture did not bind to T10B9.1A-31 that recognizes the TCR-αβ framework epitope and did not transcribe a Vδ1Cα rearrangement. Nevertheless, the subset still showed surface Vβ expression while at the same time being negative for TCR-γ. A T cell that lacks TCR-α, which is the preferred binding partner of TCR-β, can express TCR-β on its cell surface either in dimerized form with pre-Tα (72–74) or as a homodimer, as had been suggested for thymic T-cell development before pre-TCR was identified (75). Given that Vβ^+^-expressing CD4^+^ Vδ1^+^ T cells are αβT-cell progenitors, they need to ensure a controlled developmental transition beyond the DN3 stage to the DP stage, which is limited to cells that have functionally rearranged TCR-β chain genes that can pair with TCR-α. The complete lack of pre-Tα mRNA in all clones and panVδ1^+^ cultures investigated, along with the fact that TCR-δ has the same constant domain size and the same spacing of the basic residues in the transmembrane region as the TCRα chain (76), thus enabling TCR-δ to physically pair with TCR-β (60) in the absence of TCR-γ (60), suggests an intriguing scenario that does not involve a pre-TCR generation, which requires pre-Tα, i.e., the pairing of TCR-β with TCR-δ for Vδ1^+^Vβ^+^ surrogate pre-TCR.

The idea of Vδ1^+^Vβ^+^ pairing conforms with the findings of Hochstenbach et al. who described Vδ1^+^Vβ^+^ heterodimers on the CD4^+^ T-cell fraction derived from a human Vδ1^+^ Cγ^neg.^ Burkitt lymphoma (DND-41), established from pleural effusion (60). Thus, the formation of Vδ1^+^Vβ^+^ heterodimers on Vδ1^dim^ cells for surrogate pre-TCR seems completely feasible and would also make sense in terms of the quantity of pre-TCR surface expression, because the pre-TCR is expressed 50 to 100-fold lower than the TCR-αβ on mature T cells, as is the pre-B cell receptor (BCR) on the surface of pre-B cells (77). Consistently, Vδ1^dim^ cells show low Vδ1^+^ and Vβ^+^ expression. Accordingly, the Vδ1^+^Vβ^+^ heterodimer for pre-TCR would add to the list of other surrogate pre-TCRs that have been described to promote progression of DN thymocytes to the DP stage in various model systems, including γδ (78–83), αβ(84–87), αγ (88), and pTα/γ (89) heterodimers.

As Vδ1^+^Vβ^+^ pairing only occurs in the absence of the γ-chain, TCR change requires downregulation of γ-chain protein expression. Thymocytes progressively downregulate TCR-γ expression from the DN3 to the DP stage to the relatively low level found in mature peripheral αβ T cells (4). CD4^+^ Vδ1^+^ clones, correspondently to the markers that they expressed – which are consistent with DN3 thymocytes – showed a cell fraction that expressed Vδ1^+^ at low level, and were low/neg. for TCR-γ(Cγ^lo^/^neg.^). The significantly higher *in vivo* TCR-γ expression on Vδ1^+^ T cells compared to Vδ2^+^ T cells in bone marrow and peripheral blood was unexpected (Figure 10C). This observation was put into perspective during inflammation when TCR-γ expression was significantly increased in the Vδ2^+^ compared to the Vδ1^+^ T-cell subset in the peripheral blood of diseased individuals, while TCR-γ expression remained unchanged in Vδ1^+^ cells (Figure 10C).

When CD4^+^ Vδ1^+^ clone cultures were compared with γδ^+^ subsets *in vivo*, the TCR-γ expression levels of the clones (chronically exposed to inflammation) were identical to those observed in the Vδ2^+^ but not to those observed in the Vδ1^+^ subset in the blood of diseased individuals. In contrast, the CD4^+^ Vδ1^+^ clone fraction that underwent TCR change was identical to the peripheral Vδ1^+^ subset. This indicates that, in contrast to all other Vδ1^+^ cells, CD4^+^ Vδ1^+^ T cells have the capacity to modulate TCR expression, possibly due to the disulfide bond that links the constant region of Cδ to the Cγ1 segment of their TCR, a similarity they share with the Vδ2 TCR. In addition, they can downregulate TCR-γ chain expression during the process of transdifferentiation (Figure 6A).

Presuming that the Vδ1^+^Vβ^+^ heterodimer is a surrogate pre-TCR in Vδ1^lo^Cγ^neg.^ T cells, signals that trigger Vβ chain selection would require CXCR4 (90) and GATA-3 (91). Accordingly, CD4^+^ Vδ1^+^ γδ T-cell clones express high amounts of GATA-3, which allows them to control the translation of TCR-β mRNA into protein (92, 93), to increase in cell size, which is a feature that accompanies pre-TCR expression in DN3 (92, 93), and to traverse the conditional developmental arrest of the β-selection checkpoint into DN4 stage (92, 93). Moreover, GATA-3 positively regulates the transcription enhancer Eα, which is crucial for the initiation of rearrangement and expression of TCR-α (94). In this context, it was not surprising that the regulatory regions of both the TRAV-26-2 segment and the TRBV30 segment share a GATA-3-binding cis element (95). Additionally, CD4^+^ Vδ1^+^ T-cell clones simultaneously express the hallmark molecules RAG and TdT that guide thymocytes from DN3 through to the DP stage.

These results substantiate the assumption that distinctive Cβ-expressing CD4^+^ Vδ1^lo/neg.^ Cγ^neg.^ T cells, CD4^+^CD8^+^ DP Vδ1^lo/neg.^ Cγ^neg.^ T cells and TCR-αβ^+^ SP CD4^+^ or SP CD8^+^ Vδ1^lo/neg.^ Cγ^neg.^ T cells that are present in CD4^+^ Vδ1^+^ clone cultures are cellular intermediates resulting from the successful traversal of β-selection. Similar to the situation with immature CD4^+^CD8^+^-expressing “thymocytes” that express the antigen receptors and undergo positive and negative selection, which is the core process of αβT-cell development.

Our finding that newly generated CD4^+^ Vδ1^+^ clone-derived αβT cells underwent cell death when exposed to high-affinity ligands such as antibodies that specifically targeted surface Vβ chain, CD3 (soluble OKT-3), or CD3/CD28, supports the assumption that selection accompanies transdifferentiation (not shown).

Moreover – in analogy to the process of positive selection in the thymus – RAG expression was repressed in panVδ1^+^ cell cultures after the emergence of αβT cells (Figure 7C). We then verified our findings with published molecular data. The finding that TRBV30 was expressed in clones and in panVδ1 cultures as initial TCR-β chain, points to the outstanding role of TRBV30 segment regulation and function (59). The TRBV30 segment is unique as it is the only β segment located outside the main cluster, but downstream of the J and C segments and forming the 3′ end of the locus. The TRBV30 segment has the opposite transcriptional orientation to the other segments, and must therefore be rearranged by inversion of the Dβ, Jβ, and Cβ gene segments. This is in contrast to the rearrangement of all other Vβ segments – and is not deletional (59). Thus, rearrangements involving TRBV30 open up the β locus, and enable the subsequent rearrangement of other segments.

Moreover, TRBV30 segment chromatin access is biallelic (previously shown for mVβ14, the ortholog of TRBV30 in mice), and recombinational accessibility is not downregulated by TCRβ chains (96). TRBV30 transcripts were replaced by transcripts of other β rearrangements in clones and panVδ1 cultures at later time points, corresponding to the occurrence of β chains on the cell surfaces in FACS analysis. This indicates subsequent secondary rearrangements that possibly involve mechanisms earlier described for allelic inclusion and TCR revision in CD4 and CD8 αβT cells (97–100). The peculiarities in the regulation of the TRBV30 segment – independence of the elements that control access and rearrangement in the main cluster (59), an increased accessibility in thymocytes that transit from DN to DP stage in contrast to all other Vβ segments (101), and the lack of allelic exclusion and feedback inhibition (96) – has led to the assumption that the TRBV30/mVβ14 segment (59) has an entirely distinct function *in vivo*. Our data strongly support this argument.

Moreover, the *de novo* α rearrangement in Vδ1^+^ precursor cells followed the exact hierarchical order of thymocytes that begin α-locus recombination with the 3′ end Vα and 5′ end Jα segments, from where the process proceeds to the distal ends of the locus (52). PanVδ1^+^ cultures rearranged the TRAV segments TRAV-26-2, 26-1, and -24, which corresponds exactly to encyclopedic knowledge about the initiation of TCR-α locus rearrangement (52). Moreover, as these segments are interspaced in the δ locus, and removed when a functional TCR-δ1 chain is generated, *de novo* TCR-α rearrangements must occur on the chromosome where the α locus is still in germline configuration, rendering synchronous expression of TCR-α and TCR-δ possible. This does not exclude the possibility of TCR-δ genes replacing conventional TCR-α genes during the rearrangement of the α-locus, and forming hybrid Vδ1JαCα chains (67–71). VδJαCα hybrid chain transcripts were detected in few panVδ1^+^ T-cell and some CD4^+^ Vδ1^+^ clone cultures (not shown). Moreover, our data are also consistent with the view that the TCR-α locus underlies tight regulation and that the TCR repertoire is not a vast and chaotic morass, but rather a patterned and perhaps even predictable system (102). Coherently, we found the same α-chain segments rearranged in response to identical epigenetic stimuli in multiple donors, and overlapping sets of α-segments rearranged in response to slightly modulated triggers.

CD4^+^ Vδ1^+^ T cells also reveal an analogy with thymic TCR-α gene recombination, which lacks allelic exclusion but continues until a functional α chain can form an MHC-restricted TCR with the β chain (103) so that multiple V/J recombination events are able to occur on the same allele (11, 104). Likewise, the processivity that correlates with the lifespan of the precursor cell (105) was reproduced, CD4^+^ Vδ1^+^ T cells with superior viability (more than 1 year in uninterrupted culture) exhausted the α-locus. The clone-derived αβT-cell line K1.1 changed TCR months after the establishment and showed rearranged TRAV-26, -25, -14 and -3, αβ T-cell line P7 rearranged TRAV-26-2, -26-1, -24, -19, -14, -10, and -2 segment.

### Characterization of the αβT cells generated

To test functionality, the CD4^+^ Vδ1^+^-clone-derived TCRαβ^+^ T-cell lines K1.1 and P7 that lacked CD28 were examined in greater detail. These T-cell lines revealed an impaired proliferative response to mitogenic stimuli and carried public TCRs, as reported previously for viral and autoimmune diseases (Table 1) (53–59). This is consistent with the CD28^−^phenotypes’ high frequencies of functional virus-specific memory CTL that mediate TCR-mediated lysis (55). Constitutive IL-4 secretion and significant production of IFN-γ upon TCR stimulation showed that K1.1αβ^+^ and P7αβ^+^ were functional (Figure 5D).

Other CD4^+^ Vδ1^+^-clone-derived αβT cells had an effector-memory phenotype, some expressed CD28 while others did not, and a broad Vβ and Vα repertoire (not shown). They had no TRM phenotype, were negative for CD103 and were also CCR7^lo^ or negative and expressed varying amounts of CD62L and CD28 (not shown). Thus, Vδ1^+^-derived T cells had diverse but distinct cell-surface phenotypes, a complex T-cell receptor repertoire, and produced diverse cytokines. They could thus be classified as “functional” Th type cells. The TCRs sequenced were identical to viral antigen-specific TCRs (Table 1). The findings suggest that αβT cells that arise extrathymically from Vδ1^+^CD34^dim^CD4^+^ progenitors have a memory phenotype that enables them to respond rapidly to environmental challenges.

### Physiological relevance

To date the molecular determinants for thymic homing are missing, and it has been shown that neither the three-dimensional thymic microenvironment nor thymic epithelial cells are essential for T-cell development (106). Answers to the questions as to why the thymus provides a unique environment for T-cell differentiation and whether the differentiation of the broad range of TSP cells proceeds via a single canonical or via rather multiple pathways remain equally elusive (107, 108). Despite these uncertainties, the CD4^+^ Vδ1^+^ T-cell precursors show high consistency with the thymocytes’ phenotypic and functional behavior and the transition through strictly defined stages on an invariable differentiation route. The underlying genetic and physiological processes are largely identical on the molecular level, and it is evident that transdifferentiation is an efficiently controlled and thus a significant developmental pathway. Moreover, CD4^+^ Vδ1^+^ T-cell precursors – which express markers of TSP, DN1-DN3, and ISP progenitors – were as effective in generating mature αβT cells as DP thymocytes are. DP cells, constituting more than 90% of thymocytes, are selected for an MHC-restricted receptor, which is thought to occur relatively infrequently (109, 110) and results in the differentiation of only 1–2% DP precursors into mature T cells. The number corresponds exactly with what we found, namely, which 1 in 50 CD4^+^ Vδ1^+^ clones changed the TCR, and panVδ1^+^ cultures reproducibly generated 1.82% SP CD4^+^ and/or SP CD8^+^ αβT cells/panVδ1^+^ cell pool. Importantly, while homeostatic expansion reduces the complexity of the αβTCR repertoire in relation to the total number of αβT cells, CD4^+^ Vδ1^+^ T-cell transdifferentiation creates greater complexity of the αβTCR repertoire as the progenitors *de novo* generate a broad spectrum of new αβTCRs in the process of transdifferentiation. This may help assure that even centenarians can acquire immunity to newly encountered antigens.

Moreover, elevated precursor numbers and Vδ1^+^Cβ^+^ transdifferentiation intermediates were found in the body fluids of inflamed tissue (peripheral blood, pleura, inflamed joints, and ascites), which is in line with the observation that the number of Vδ1^+^ γδT lymphocytes is expanded in human diseases, including infections (111–114), but also rheumatoid arthritis (115), multiple sclerosis and HIV (116). This indicates that transdifferentiation is a highly economical process that only takes place in inflamed tissues that require T cells with diverse and adaptive TCRs. The high consistency with thymocytes in terms of the developmental route and productive efficacy thus suggests that the replenishment of the peripheral αβT-cell pool through Vδ1^+^-descendants is one strong principle in T-cell homeostasis.

In summary, this study describes the unique, previously unknown role of peripheral CD4^+^ Vδ1^+^ γδT cells as αβT cell precursors that can respond to hematopoietic stressors such as inflammation by differentiating into functionally, mature αβT cells at the site required. We describe the expression of HSC and progenitor markers as this subset’s peculiarity. We pinpoint the transdifferentiation of CD4^+^ Vδ1^+^ γδT cells as a process of TCR re-organization that is embedded in a developmental route similar to thymic αβ T-cell development but distinguishable from the latter by Vδ1^+^Vβ^+^ Cγ^neg^Cα^neg^ intermediates, which suggests the formation of Vδ1^+^Vβ^+^ heterodimers for surrogate pre-TCR. The conclusions drawn from the *in vitro* data are strongly supported by the results of *ex vivo* analyses of diverse body fluids, where the progenitor’s Cβ-expressing Vδ1^dim^Cγ^lo/neg^ transdifferentiation intermediates were detected in inflamed tissue.

Most importantly, the study provides a conceptual framework for a central goal of (developmental) immunology, namely, to understand how T-cell development is ultimately conducted in the absence of thymic function. The assignment of this fundamental role for γδT cells opens a new vista in immunology and requires reevaluation of adaptive immune responses in infection, autoimmunity and cancer.

## Materials and Methods

All procedures were carried out according to the Declaration of Helsinki and were approved by the Clinical Ethics Committee at the University of Tübingen (projects 38/2009B02 and 470/2013B02).

### Seven-color flow cytometry

Cells were pretreated with FcR Blocking Reagent (Miltenyi Biotec) according to the manufacturer’s instructions, resuspended in flow cytometry buffer [PBS, 2% (vol/vol) FCS, 250 mM EDTA] and incubated for 15 min (8°C, in the dark) with one or more of the following monocolonal antibodies, all specific for human epitopes: FITC-anti-Vδ1, unconjugated anti-Vδ1 (TS8.2, Fisher Scientific), APC-anti-TCRγδ (B1, BioLegend), PE-anti-TCRγδ (recognizing Cγ; 11F2, BD Pharmingen), PE-anti-TCRγδ (recognizing Cδ; SA6.E9, Life Technologies) PE-anti-TCRαβ (T10B9.1A-31, BD Pharmingen), FITC-anti-TCRαβ (WT31, BD Pharmingen), murine anti-human TCR-αβ mAb (BMA 031, Behringwerke Marburg). PE-anti-Vδ2 (B6, BD Pharmingen), FITC/PE/PerCP-anti-CD4 (SK3, BD) or Vioblue-anti-CD4 (VIT4, Miltenyi Biotec), FITC/PE/PerCP/APC-anti-CD3 (SK7, BD) APC-anti-CD3 (MEM57, Exbio), APC-anti-CD8β (2ST8.5H7, BD), and APC-H7-anti-CD8α (SK1, BD). FITC/APC-anti-CD62L (LT-TD180, ImmunoTools), PE/APC-anti-CD25 (2A3, BD Pharmingen), PE-anti-CD135 (4G8, BD Pharmingen), PE-anti-CD127 (M21, BD Pharmingen), and APC-anti-CD132 (4G3, BD Pharmingen). All IgG isotypes and secondary antibody rat anti-mouse IgG 1-PE (X56) were purchased from BD Pharmingen. Cytokines were detected by intracellular staining using standard procedures, and with APC-conjugated anti-IL-2 (MQ-1-17H12), -IL-4 (MP4-25D2), -IL-5 (TRFK5), -IL-10(10JES3-19F1), -IL-13(JES10-5A2), -TNF-α (MAb11), -IFN-α (B27), all from BD Pharmingen, PE-anti-IL-17A from BioLegend, and biotinylated-anti-TGF-β (MAB240) from R&D Systems. Streptavidin conjugated with APC or PE (Invitrogen) was used for the detection of biotinylated antibodies; the cells were transferred into new vials after each washing step in order to reduce background and exclude false positive results.

In order to exclude dead cells from being analyzed, all cells were subject to live/dead exclusion using the Alexa Fluor 350 NHS Ester kit. In order to exclude the possibility of contaminants from feeder cells, irradiated feeder cells were cultivated and analyzed as clone cultures and used as controls in all experiments performed. Cells were analyzed using LSR II or FACS Calibur systems, and the FACS Diva©software and CellQuest software programs were used for the acquisition and analysis of flow cytometric data.

TRBV repertoires were analyzed with the IOTest^®^ Beta Mark Kit (Beckman Coulter), a multi-parametric analysis tool designed for the quantitative flow cytometric determination of the TCR Vβ repertoire of human T lymphocytes. Taking advantage of the fact that Vβ specificities may be grouped into mutually exclusive combinations, three Vβ expressions can be detected in the same tube using an innovative staining strategy that uses three mAb stained with two fluorophores only. One mAb is conjugated to a FITC molecule, the second to PE and the third one is a carefully balanced mixture of a PE- and a FITC-conjugated form.

### Immunofluorescence

Vδ1^+^ CD4^+^ T-cell clones were harvested from culture dishes and washed twice in sterile 1 × PBS. For TCR-β chain expression analysis, cells were applied to the slides by cytospin, fixed in −20°C acetone/methanol (50/50; 2 min), and stained with mouse-anti-human TCR Beta F1 (1:50) (8A3, Thermo Scientific). Cy™ 3-goat-anti-mouse IgG (1:400) (Jackson ImmunoResearch) was used to visualize TCR-β chain expression. Slides were analyzed with an Axiophot microscope (Zeiss) and the AnalySIS^®^ 3.2 software (Soft Imaging Systems).

### Molecular methods

#### RNA isolation/cDNA synthesis

RNA was isolated using the RNeasy Plus Mini Kit (Qiagen). cDNA was synthesized using the Superscript III First Strand Synthesis Super Mix formulation (Invitrogen). All cDNAs were tested for the expression of a 800 bp amplicon of β-actin.

#### Real-time PCR

cDNAs derived from CD4^+^ Vδ1^+^ clones and from panVδ1^+^ cultures were analyzed with ABI TaqMan primer/probe sets for PTCRA (Hs00300125_m1), GAPDH (Hs02758991_g1), and RORC (Hs01076122_m1), all purchased from Life Technologies. All other primers are self-designed and are available upon request. Invitrogen’s TaqMan assay reagent and BioRad’s IQ Master Mix were used for qPCR. Gene expression was calculated using the change-in-threshold method [ΔC_(T)_] and GAPDH as reference.

#### TRAV chain analysis

Done in accordance with Han et al. (117).

#### TRBV spectratyping

Performed according to Gorski et al. (118) with minor modifications. 5′FAM-labeled BC primers were used, PCR amplicons were detected using an ABI 3130xl Genetic Analyzer, the GeneScan 600 LIZ dye size standard, and the GeneMapper software (both Applied Biosystems).

#### γδ immunoscope

Performed with discriminating primers obtained from Annik Lim and according to her protocol (personal communication).

#### Identification of TCR-CDR3 regions

The relevant PCR products required for spectratype analysis were reamplified with unlabeled C primer. Sequencing was performed with the 3130xl Genetic Analyzer (Applied Biosystems) using the BigDye Terminator v3.1 Sequencing Kit according to the manufacturer’s protocol. Translation of the cDNA sequence into the protein sequence was conducted with the EMBOSS Transeq software, which is available free of charge (119, 120).

### Cell culture, selection, and cloning experiments

Informed consent was obtained from all volunteers. Sterile heparinized peripheral blood was collected from normal, healthy donors (*n* = 12); healthy bone marrow were leftovers from HLA-typing of potential stem-cell donors (*n* = 10), aliquots of LPH were leftovers from quality control after graft generation (*n* = 12). All samples were obtained and handled according to the Declaration of Helsinki and the procedures were approved by the Clinical Ethics Committee at the University of Tübingen. PBMCs were isolated by density centrifugation (Ficoll-Hypaque). Up to 2 × 107 PBMCs were pretreated using FcR Blocking Reagent (Miltenyi Biotec) according to the manufacturer’s protocol, stained with FITC-anti-Vδ1 (TS8.2, Fisher Scientific) using 10 μl antibody per 1 × 107 PBMCs (15 min, +8°C, in the dark). Subsequently, the Anti-FITC MultiSort Kit (Miltenyi Biotec) was used for the isolation of “dim” cells. The cells were separated with columns (Miltenyi Biotec). FACS analysis usally had a purity of >99% Vδ1^+^ T cells and less than 0.16% αβTCR^+^ cells. Alternatively, Vδ1^+^ γδT cells were selected with the Anti-TCRγ/δ MicroBead Kit (Miltenyi Biotec).

For the establishment of Vδ1^+^CD4^+^ T-cell clones, Vδ1^+^ T cells were enriched for CD4 using the Dynal^®^ CD4 Positive Isolation Kit (Invitrogen). All procedures were carried out according to the manufacturer’s instructions. Vδ1^+^ CD4enriched T cells were cloned using the limiting dilution procedure (0.1 cells/well) in round bottom 96-well plates in the presence of 200 IU/mL IL-2 (ImmunoTools), 1.0 ng/mL IL-7 (ImmunoTools), 0.25 μg/mL phytohemagglutinin (Oxoid), and 1 × 105 allogeneic, 80 Gy-irradiated feeder cells. After 21 days, Vδ1^+^ CD4^+^ T-cell clones were identified by FACS analysis and kept under standard culture conditions – which represents mild inflammation – until further manipulation and analysis. Cloning strategy routinely yielded 5–10% of initially seeded Vδ1^+^CD4^+^ T cells (0.1 cells/well). For panVδ1 culture assays, 1,000 Vδ1^+^ cells were seeded per well in round bottom 96-well plates and cultured in RPMI 1640 supplemented with 10% heat-inactivated FCS, L-glutamine (4 mM), and penicillin (50 U/mL)/streptomycin (50 μg/mL), all purchased from Biochrom GmbH.

Overt inflammation was mimicked in panVδ1 cultures by adding the following cytokines to the standard culture medium in week 3: IL-1β (10 ng/mL; ImmunoTools), IL-18 (10 ng/mL; R&D Systems), IL-6 (50 ng/mL; ImmunoTools), sIL-6R (100 ng/mL; ImmunoTools), and IL-12 (10 ng/mL; ImmunoTools). Monocytes that had been preactivated for 3 days in the presence of these inflammatory cytokines were also added to the culture. Monocytes were generated from PBMNCs by plastic adherence in cell-culture flasks for 2 h (PBMNC density 1.5 × 106/mL in RPMI 1640 standard medium; 5% CO_2_, water-saturated atmosphere, 37°C). Non-adherent cells were subsequently removed along with the supernatant; adherent cells were washed twice with warm PBS, and new culture medium containing inflammatory cytokines was added. After 3 days, the monocytes were removed from the bottom of the culture flask with a cell scraper, counted, centrifuged, resuspended in conditioned medium, irradiated (80 Gy), and 1 × 105 monocytes/well added to panVδ1^+^ cell cultures.

### Functional analysis

αβT cells derived from transdifferentiated Vδ1^+^ CD4^+^ γδ T-cell clones were stimulated for 5 h with PMA (50 ng/mL) and ionomycin (750 ng/mL, Sigma). Brefeldin A (10 μg/mL) was added for the last 60 min of incubation. Cytokine production was measured by intracellular staining and FACS analysis as described above.

### Statistical analyses

Statistical analyses were performed with the GraphPad Prism software V5.0 (GraphPad Software). Statistical differences were analyzed using the parametric student *t*-test, error bars in the graphs depict the SEM. A *p* < 0.05 was considered as statistically significant.

## Author Contributions

Hendrik Ziegler, Christian Welker, and Marco Sterk contributed equally to the work, and performed most of the experiments, contributed to and established methodology; Jan Haarer contributed and established methodology, and performed experimental work; Hans-Georg Rammensee and Rupert Handgretinger contributed to experimental design and Rupert Handgretinger provided essential material. Christian Welker prepared all figures and helped editing the manuscript; Karin Schilbach initiated all work on Vδ1^+^ CD4^+^ cells, proposed and identified Vδ1^+^ CD4^+^ cells as a CD34^+^ T cell precursor, planned, designed, supervised all experiments *in vitro* and *ex vivo*, interpreted data, identified transdifferentiation as the re-organization of the TCR-γδ into TCR-αβ via a Vδ^+^Vβ^+^ Vγ^neg.^Vα^neg.^ intermediate, established the developmental scheme, and wrote the manuscript.

## Conflict of Interest Statement

The authors declare that the research was conducted in the absence of any commercial or financial relationships that could be construed as a potential conflict of interest.

## Supplementary Material

The Supplementary Material for this article can be found online at http://www.frontiersin.org/Journal/10.3389/fimmu.2014.00645/abstract
