# Supplementary figures and images for: Human Peripheral CD4+ Vδ1+ γδT Cells Can Develop into αβT Cells
Source: Front Immunol. 2014 Dec 17;5:645. doi: 10.3389/fimmu.2014.00645 (PMC4329445; doi:10.3389/fimmu.2014.00645)

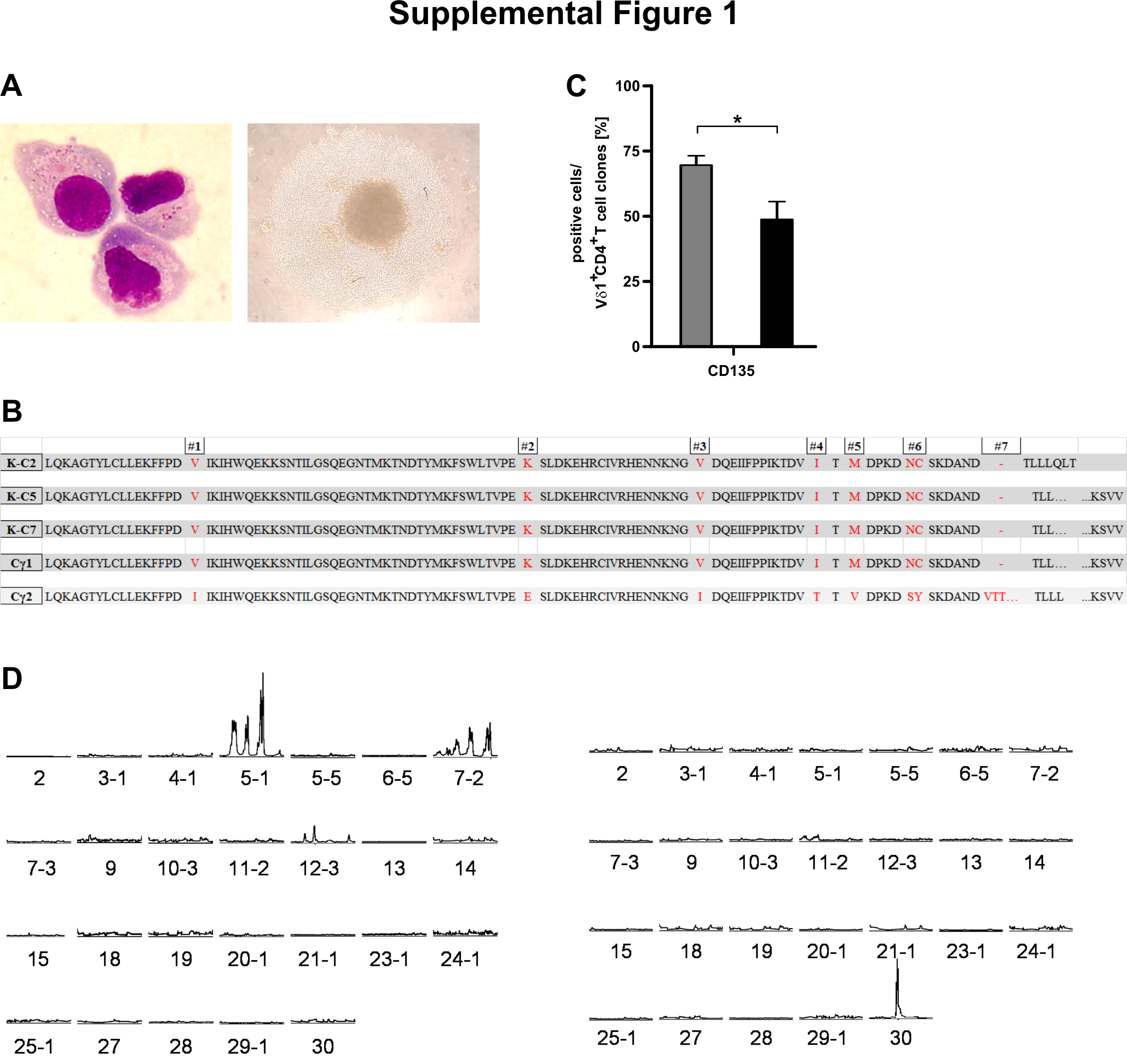

Supplement: Supplementary file 1 [file Image_1.TIF]

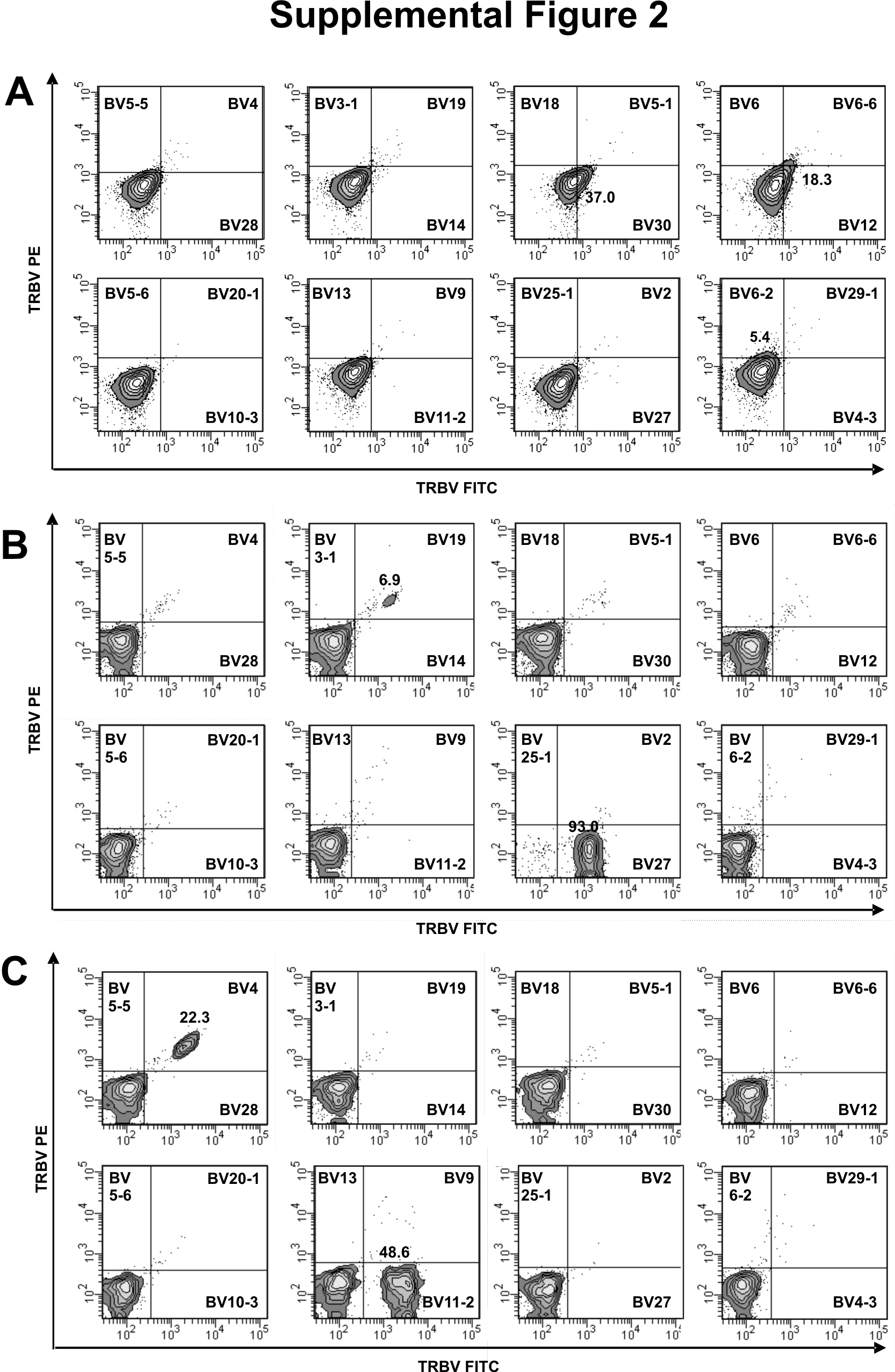

Supplement: Supplementary file 2 [file Image_2.TIF]
